# Identifying Social factors that Stratify Health Opportunities and Outcomes (ISSHOOs) in pain research: consensus recommendations for the collection and reporting of equity-relevant data

**DOI:** 10.1016/j.eclinm.2025.103586

**Published:** 2025-10-24

**Authors:** Emma L. Karran, Aidan G. Cashin, Alessandro Chiarotto, Saurab Sharma, Trevor Barker, Mark A. Boyd, Lara J. Maxwell, Vina Mohabir, Jennifer Petkovic, Peter Tugwell, G Lorimer Moseley, ISSHOOs Group, Oluwafemi K. Ajayi, Ruth P. Appiah, Cheryl Barnabe, Sónia F. Bernardes, Didier Bouhassira, Margarita Calvo, Mary Cowern, Brona M. Fullen, Catherine Hofstetter, Mary R. Janevic, Flavia P. Kapos, Dale J. Langford, Bronwyn Lennox–Thompson, John D. Loeser, Tonya Palermo, Romy Parker, Karma Phuentsho, Andrew SC. Rice, Sinan Tejani, Rolf-Detlef Treede, Janice Tufte, Angela Yeo

**Affiliations:** a The ‘Identifying Social Factors that Stratify Health Opportunities and Outcomes’ (ISSHOOS) Collaboration Core Research Group; b IIMPACT in Health, University of South Australia, Kaurna Country, Adelaide, South Australia, Australia; c Centre for Pain IMPACT, Neuroscience Research Australia, Sydney, New South Wales, Australia; d School of Health Sciences, Faculty of Medicine and Health, University of New South Wales, Sydney, New South Wales, Australia; e Department of General Practice, Erasmus MC, University Medical Center, Rotterdam, the Netherlands; f Pain Management and Research Centre, Royal North Shore Hospital, Northern Sydney Local Health District, Sydney, New South Wales, Australia; g Pain Management Research Institute, Kolling Institute, Faculty of Medicine and Health, The University of Sydney and Northern Sydney Local Health District, Sydney, New South Wales, Australia; h Faculty of Health and Medical Sciences, University of Adelaide, Adelaide, South Australia, Australia; i Northern Adelaide Local Health Network, South Australia, Australia; j Ottawa Hospital Research Institute, Methodological and Implementation Research, Ottawa, Canada; k Faculty of Medicine, Department of Medicine, University of Ottawa, Ottawa, Canada; l Child Health Evaluative Sciences, Peter Gilgan Centre for Research and Learning, The Hospital for Sick Children, Toronto, Ontario, Canada; m WHO Collaborating Centre for Knowledge Translation and Health Technology Assessment in Health Equity, Bruyere Health Research Institute, Ottawa, Canada; n Department of Medicine, Faculty of Medicine, University of Ottawa, Ottawa, Canada; o University of Ottawa, School of Epidemiology and Public Health, Faculty of Medicine, Ottawa, Canada

**Keywords:** Health equity, Pain, Recommendations, Reporting, Social determinants of health, Sociodemographic characteristics

## Abstract

**Background:**

The aspiration to improve health equity is fundamental to scholarly focus and action in public health, and highly relevant to addressing the global burden of pain–the leading contributor to disability worldwide. There is potential for advancement towards health equity to be facilitated by greater access to data that identifies the role of socio-demographic factors in pain and health outcomes.

**Methods:**

The ‘Identifying Social factors that Stratify Health Opportunities and Outcomes (ISSHOOs) in Pain Research’ project was a multi-stage process that aimed to reach consensus on the most important equity-relevant items to include in all human adult pain research. Conducted April 2022–May 2025, it incorporated two scoping reviews (published 2023), an international Delphi study (published 2025), consensus meetings and focus groups; prioritising global participation, patient perspectives, and interdisciplinary expertise throughout.

**Findings:**

Three hundred and four individuals from 45 countries, across six continents, contributed to developing two sets of items. Set A, the ‘minimum dataset’, is a globally relevant set of eight standardised socio-demographic items (age, sex, gender identity, place, race/ethnicity/cultural identity, education, financial position, work status), accompanied by concise guidance to assist implementation and setting-specific tailoring; Set B is an ‘extended dataset’ of optional items from which researchers can select items consistent with their study population and research questions. The ISSHOOs recommendations offer a culturally sensitive, cross-culturally relevant, practical and highly useful resource.

**Interpretation:**

Routine adoption and clear reporting of the ISSHOOs datasets across all human adult pain research will lead to improved and harmonious descriptions of research participants across health equity domains. Our goal is to promote equity-relevant awareness and understanding, and ultimately drive progress towards reducing avoidable disparities in health for people with pain, with potential for broader application to other fields of health.

**Funding:**

10.13039/501100000925NHMRC (Australia); 10.13039/100001744MAYDAY Fund; IASP; Canada Research Chair Program; European Horizon 2020 Research and Innovation Programme; ZonMw programs.

**Research in context**

**Evidence before this study**

Socio-demographic data are inconsistently and inadequately reported in health research. This confounds interpretations related to the generalisability of research findings, limits exploration, and perpetuates the invisibility of health inequities. Improved data collection and reporting practices that adequately and consistently reveal the characteristics of those included in studies—also allowing attention to be drawn attention to *who is not included*—is an urgent and important need.

**Added value of this study**

The ‘Investigating Social factors that Stratify Health Opportunities and Outcomes (ISSHOOs) in Pain Research’ project addresses this critical need to improve and standardise data collection and reporting practices in the pain field. More than 300 experts from 45 countries participated in a multi-stage consensus process that prioritised global partnerships, the perspectives of people with lived experience and interdisciplinary collaboration. Two sets of standardised items were finalised: a ‘minimum dataset’ (Set A)—recommended for use in all human adult pain research; and an ‘extended dataset’ (Set B) of optional items for consideration. The ISSHOOs recommendations offer a culturally sensitive, cross-culturally relevant and practical resource to facilitate comprehensive, harmonious, data collection and reporting of socio-demographic factors in all human adult pain research.

**Implications of all the available evidence**

Through widespread uptake, the ISSHOOs recommendations will facilitate progress towards understanding and addressing health inequities for people with pain. Moreover, they have potential for broader application to other fields of health.

## Introduction

Health equity—the state in which everyone has a fair and just opportunity to be as healthy as possible,^1^ is a guiding principle of initiatives to tackle disease and improve public health. An equity focus prioritises addressing avoidable, unfair and systemic factors that underpin disparate health outcomes between population groups. The imperative to address health inequities was highlighted by the report of the World Health Organisation (WHO) Commission on Social Determinants of Health (2008).^2^ Subsequent efforts to improve equity considerations in health research have led to the development of ‘equity extensions’ to methods-specific reporting guidelines, and equity-adaptations of the widely used PRISMA (systematic reviews) and CONSORT (trials) checklists.^3^, ^4^, ^5^ Despite these efforts, substantial improvements in the reporting of factors relevant to evaluating health equity are lacking—posing a significant barrier to advancing awareness, understanding and action.

There is increasing recognition that health researchers must do better at collecting, reporting and interpreting ‘equity-relevant’ data.^6^, ^7^, ^8^, ^9^ The absence of key socio-demographic data confounds interpretations related to the generalisability of study findings, limits exploration, perpetuates the ‘invisibility’ of health inequities, and impedes progress towards understanding and addressing health inequities. Furthermore, research participants typically do not represent the broad range of socio-demographic characteristics of the general population, nor those who experience poorest health: people who experience disadvantage and marginalisation are commonly under-represented. Improved data collection and reporting practices in health research—adequately revealing who is in studies (and who is not)—is an urgent and critical need.

Pain is the leading cause of disability worldwide and imposes vast global burden affecting individuals, healthcare systems and economies.^10^ Chronic pain is listed in the current WHO work plan (GPW14) as warranting equity-relevant attention to reach benchmarks outlined in the Sustainable Development Goals.^11^ Certainly, the role of social determinants of health are as relevant to pain as they are to other diseases that are unequally distributed in society,^12^^,^^13^ and the need to confront the lack of diversity in pain research is well recognised.^14^ Routinely collecting and reporting data that describe study participants across socio-demographic, economic and environmental factors known to relate to health and health equity in a standardised manner, will offer enormous opportunity to progress the field.^15^ Such data may be used for subgrouping (e.g. to determine differential effects of interventions across strata of society); determining generalisability and transportability; assisting adherence to equity-reporting guidelines; and facilitating data-pooling across research studies.

In pain research and in broader spheres of health there is well-aligned recognition of the need for harmonised data collection that can lead to improved clinical and public health evidence-based decision making, with greater understanding and action on health equity an over-arching aspiration. The development of consensus-based recommendations to guide standardised data collection and reporting in pain research also has the potential to yield findings that can be usefully applied to other health fields.

The aim of the ‘Identifying Social factors that Stratify Health Opportunities and Outcomes (ISSHOOs) in Pain Research’ project was to develop a standardised set of equity-relevant items (i.e. questions and response-categories) that can be routinely collected and reported in all pain research involving human adults. This research includes a wide range of fields in which pain is the primary problem or an outcome of a disease (e.g. rheumatology and musculoskeletal health, cancer, anaesthesiology). Our goal was to produce a useful and practical data collection tool that is globally and cross-culturally relevant, adaptable, and freely accessible. The ISSHOOs recommendations will also provide funding agencies, journal editors and peer reviewers with a benchmark from which to evaluate research proposals and reports (including suitability for publication)—thus improving the quality, relevance and impact of research across the field. In this paper we describe the methods underpinning the ISSHOOs recommendations and present the ISSHOOs recommendations, inclusive of guidance for context-specific tailoring and implementation.

## Methods

**Fig. 1:**
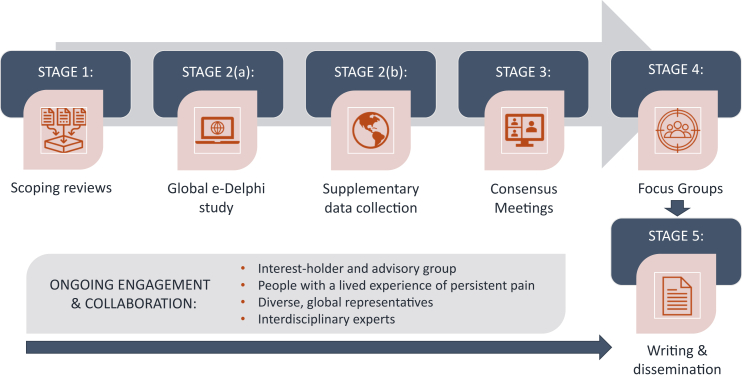
**Overview of the five main stages of the ISSHOOs project**. Stage 1—Scoping Reviews to identify relevant items used in healthcare settings and research; Stage 2(a)—Delphi study to reach consensus on important items for consideration; Stage 2(b)—Supplementary Data specifically targeting under-represented groups; Stage 3—consensus on the ISSHOOs item sets (A and B); Stage 4—focus groups, item refinement; Stage 5—writing and dissemination.

The process to develop the ISSHOOs Recommendations was informed by the Enhancing Quality and Transparency of Health Research (EQUATOR) methodological framework for the development of reporting guidelines (Appendix 1).^16^ The overarching protocol of the ISSHOOs project was registered in Open Science Framework (https://osf.io/dqan2/) and has been published^17^; minor deviations from this protocol are described in Appendix 2. The University of South Australia Human Research Ethics Committee approved all stages of the project separately (as required) prior to commencing the research, and all participants gave informed consent before participating. In this section we provide an overview of the development of the ISSHOOs recommendations. The five main stages of the ISSHOOs project are illustrated in Fig. 1.

### Participants

**Fig. 2:**
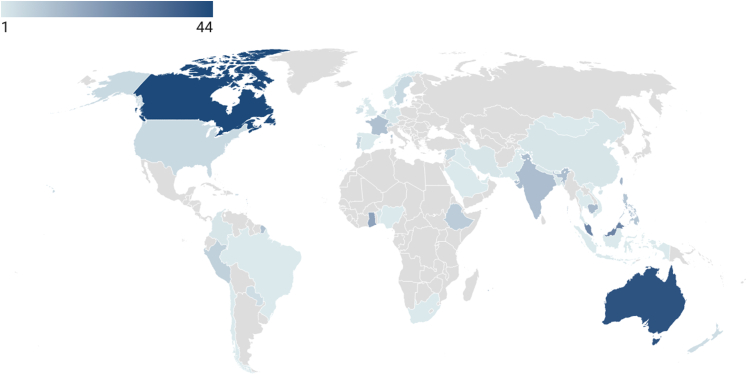
Global distribution of participants and collaborators.

**Fig. 3:**
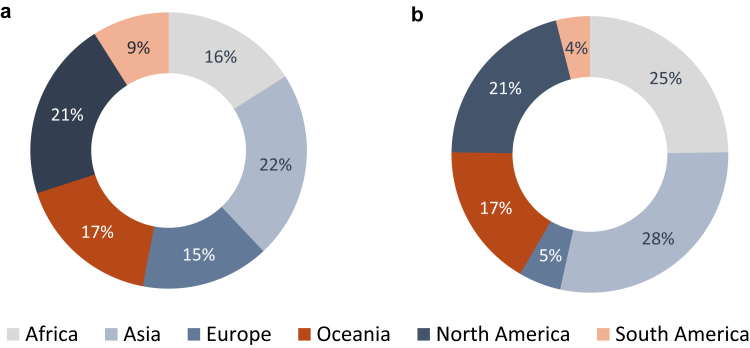
(a) Geographic distribution of all participants and collaborators (n = 301). (b) Geographic distribution of all participants and collaborators with a lived experience of persistent pain (n = 139).

The ISSHOOs Core Research Group (CRG) (11 members) was established in 2022 and includes people with expertise in pain, health equity, interest-holder engagement, consensus methods and/or a lived experience of persistent pain. Formal engagement with an appointed 21-member ‘Interest-holder and Advisory Group’ (IAG) commenced in February 2023 (see Appendix 3). ‘Interest-holders’ were defined as those with interests in improving outcomes for people with pain; those with responsibility for pain (or health)-related decisions and those affected by these decisions.^18^ Members include individuals from six continents, pain journal editors, clinicians, patients, and representatives from national, regional and global pain associations and international funding organisations. Overall, 304 individuals have contributed to this project. They included individuals from all of the WHO regions (see Figs. 2 and 3 for the geographical distribution of participants), and highly diverse–spanning a wide range of socioeconomic backgrounds, ages, languages, cultures, religions, sexual orientations and residential locations. People with a lived experience of persistent pain contributed to all project stages from conception to dissemination (see Box 1 for Summary of Patient and Participant Involvement (PPI)).

Box 1 **Summary of Patient and Participant Involvement (PPI) according to the GRIPP2-SF reporting checklist19**

**Table:** 

**Aim**	To develop a set of standardised items to facilitate the routine collection and reporting of ‘equity-relevant’ data in all human adult pain research. To collaboratively involve patients and public as research partners throughout the project, and as participants in project stages 2, 3 and 5.
*The CRG & IAG involved people with lived experience of persistent pain who contributed to identifying the need for this work, refining the focus & developing the project objectives.*	
**Methods**	The CRG involved 2 patient partners who participated in conceptual planning, protocol development, recruitment, analysis and interpretation, group discussions, study reporting, and dissemination planning. They also worked with the researchers to ensure that communication was inclusive, appropriate and understandable to a ‘lay’ audience. They provided critical revision of the content of this manuscript and are co-authors. The IAG involved eight (additional) patient-research partners who provided oversight via contributions in online meetings and correspondence via email. All reviewed study protocols, assisted Delphi study recruitment, participated in the Delphi study, and provided feedback on the final ISSHOOs items.
*Patients were collaboratively involved as research partners in all stages of this project.* *The Delphi study, supplementary data survey & focus groups involved >50% participants with a lived experience of persistent pain. All patient research partners were reimbursed.*	
**Results**	PPI contributed to this project and results in many different manners, including: • Finalising the selection of items for Delphi round one. • Participating in the Delphi study. PPI data were disaggregated and considered separately in all analyses. These data contributed important contextual insights, with impact on item inclusion in subsequent Delphi rounds. • Completing the ‘supplementary data’ survey. Representation from diverse, ‘hard-to-reach’ groups informed identification of the ‘most important’ items to include. • Participating in focus group discussions • Assisting with refining the final item sets and drafting the project outputs.
*Results will be disseminated* via *manuscripts and conference presentations and shared with study participants* via *email, including a website link (*www.isshoos.org*). The authors will seek endorsement & promotion from journal editors, funding agencies, pain associations and professional associations in fields in which pain outcomes are of primary concern (*e.g. *rheumatology, rehabilitation, surgical, cancer).*	
**Discussion and Conclusions**	PPI was integral to this project and highly important for the achievement of the objectives, including the development of two item sets that seek to be globally relevant and cross-culturally acceptable. Factors related to successful engagement included involving people with extensive experience in interest-holder engagement in our research team and having a global network of collaborators who facilitated connections. Patient-research partners were involved from the beginning, which fostered committed, collaborative relationships and offered valuable learnings to guide communication strategies (particularly related to clarity, readability, and inclusivity). Limitations of PPI in the context of this study related to the challenges of including people from diverse backgrounds and settings, from historically marginalised/minoritised groups, and adults who experience socio-economic disadvantage.
**Reflections**	We prioritised active PPI and collaboration throughout all stages of this project. Our most important challenge was to seek the perspectives of typically under-represented groups. We developed a targeted recruitment strategy to achieve greater diversity in included perspectives (via the ‘supplementary data’ survey) which was an important adaptation to our protocol. We acknowledge however, persisting limitations related to the representativeness of our study cohort in the global context.

**Abbreviations:***PPI,* Patient and Participant Involvement; *CRG,* Core Research Group; IAG, Interest-holder and Advisory Group.

### Project stages

#### STAGE 1: Scoping reviews

We conducted two scoping reviews to explore current approaches to collect data relating to the social determinants of health. The (combined) review protocol was pre-registered (https://osf.io/dqan2/); these manuscripts have been published elsewhere.^20^^,^^21^ In the first review,^20^ we searched academic databases from 1/01/2010 to 3/05/2022. We reported the content of social needs screening tools that have been developed for use in clinical settings, including details of how these social needs were screened. In the second review,^21^ we identified what and how data relating to the social determinants of health were collected in 200 recent, equity-relevant studies, undertaken in any field of health during the most recent calendar year (2021). We extracted the items (question-and-response-sets) from both reviews and mapped them to the ‘PROGRESS-Plus’ framework.^22^ PROGRESS is an acronym (**P**lace; **R**ace, ethnicity, culture and language; **O**ccupation/work status, **G**ender and sex; **R**eligion, **E**ducation, **S**ocio-economic status, **S**ocial capital) that offers a highly useful approach for identifying and classifying a broad range of equity-relevant data. ‘Plus’ incorporates additional context-specific characteristics that can contribute to inequities in health including age, disability, and sexuality. The ISSHOOs CRG identified themes within the PROGRESS-Plus categories and reached full agreement on items that best represented each sub-category through iterative discussion. In addition, where CRG members were aware of relevant items that had previously been produced through consensus processes (e.g. standardised items for collecting self-reported data on gender, race and ethnicity^23^), or items that were currently recommended by interest-holder-engaged advocacy groups (e.g. ACON (previously AIDS Council Of New South Wales, Australia) gender and sexuality indicators^24^)—we included these items. A set of 43 items were consolidated for inclusion in round one of the following Delphi study (Appendix 4).

#### STAGE 2(a): Delphi study

The protocol for the three-round Delphi study was approved by the University of South Australia Ethics Committee (ID 204295) and pre-registered (https://osf.io/knm8q/). The study was conducted between the 5th of June 2023 (opening of round one) and the 6th of November 2023 (closing of round three). The Delphi Study has been reported in accordance with the ACCORD (ACcurate COnsensus Reporting Document) checklist and published elsewhere–we refer readers to this manuscript^25^ for full details. We invited an international panel of experts to participate in a three-round e-Delphi process, based on their expertise in pain, social determinants of health, health equity, or a lived experience of persistent pain. We aimed to recruit diverse, global participants in the Delphi study and offered to translate all research tools into other languages as required (as guided by our recruitment networks). The Delphi study was translated into Spanish using Google Translate, checked for accuracy and meaning by a bilingual investigator, and completed in Spanish by three participants. In round one, 168 participants (35% with ‘lived experience’) from six continents rated the importance of including each of the initial 43 items in a ‘minimum dataset’ of equity-relevant items. Twenty-nine items reached agreement for inclusion in round one (based on a threshold of panel median of ≥7 on a 9-point agreement scale); no items were excluded. Participants suggested additional items, which were collated by the CRG, and 21 new items were proposed. In Round two, 152 participants (90% of Round one) voted on 35 items (the new items and 14 items that were ‘uncertain’ from round one), 25 of which reached agreement for inclusion. In Round three, 142 participants (93% of round two) prioritised (within each PROGRESS-Plus category) the 54 items that reached the threshold for inclusion. Participants also rated the importance of including one or more items from each PROGRESS-Plus category in the minimum dataset.

In all analyses, we disaggregated the data to explore the opinions of people with lived experience of persistent pain, and (where possible) the opinions of people with experiences of relevant disadvantage, in addition to analysing ‘whole group’ data. Our analyses indicated that including items related to ‘religion’ was considered comparatively less important than including items from the other PROGRESS-Plus categories. At the item-level, we obtained data to inform the relative importance of items within each category for subsequent consideration and discussion at the Consensus Meetings.

#### STAGE 2(b): Supplementary data survey

Following input from the IAG we identified the need to seek further perspectives from people who reside in diverse settings and/or experience disadvantage or marginalisation—i.e. we sought to address the highly common problem of under-representation of ‘hard-to-reach’ groups in health research.^26^ Specifically, we aimed to include people who met one or more of the following criteria: low educational background; low socioeconomic position; belonged to a minoritised/marginalised group (e.g. due to cultural identity, gender, sexual orientation, religion); resided outside of major cities, and (predominantly) from outside of North America and Australia. Individuals who were likely to meet one or more of these criteria were identified and recruited through the personal contacts of research team members and their extended networks. Participants were invited to complete a modified version of the round three Delphi survey, which was translated as required.Fifty-five participants agreed to participate in this research and completed the online survey; 44 surveys were completed in English and 11 surveys were completed in Arabic. The translated surveys were produced using Google Translate and reviewed for accuracy and meaning by a bilingual investigator. Participant characteristics are summarised in Appendix 5. We analysed ‘whole group’ data and disaggregated data (according to type of potential disadvantage) and explored inconsistencies between the supplementary data and the results of the Delphi study. Consistencies, inconsistencies, and insights were prepared for presentation and consideration at the Consensus Meeting.

#### STAGE 3: Consensus meeting process

The ISSHOOs CRG identified the need for two sets of items—Set A and Set B—which were addressed in two separate Consensus Meetings. An overview of the distinction between the two item sets is provided in Box 2. Thirty individuals participated in the consensus process: the ISSHOOs CRG (11 members), a sub-group of the IAG (15 members) and seven additional contributors who were invited due to their relevant expertise and/or global perspectives (see Appendix 6 for the participant list). The meetings were conducted online during March and August 2024.

Box 2 **Overview of the ISSHOOs datasets: recommended for use in all human adult pain research**

**Table:** 

Set A: Minimum Dataset (Descriptive Items)	
*Researchers recommended to consider including ALL items in Set A*	
**Overview**	Set A will enable participants to be described across a range of socio-demographic characteristics known to relate to health & health equity
**No of items**	8 *recommended* items
**Included**	*For researchers:* Brief information and rationale; guidance for tailoring, development or omission of items *For participants:* Brief introductory information and item-specific preambles
**Purpose**	Data will assist identification of study generalisability, enable sub-group analyses & data pooling, inform research interpretations, prompt equity considerations throughout the research process
Set B: Extended Dataset (Contextual items)	
*Researchers recommended to consider selecting a SUB-SET of items from Set B, relevant to their study population, setting & research questions*	
**Overview**	Set B items predominantly identify contextual factors that influence health and are relevant to health equity
**No of items**	30 *optional* items for consideration
**Included**	*For researchers*: information to guide item selection and tailoring
**Purpose**	Data have the potential to prompt equity-relevant awareness, increase understanding, facilitate analyses of treatment effect modifiers, and identify social or environmental factors as targets for interventions

Consensus Meeting #1: The goal of this meeting was to reach consensus on the Set A items. Participants were emailed ‘*Essential Pre-Meeting Reading’* which summarised relevant background, outlined the goal, structure and ‘ground rules’ of the meeting, detailed ‘Essential Criteria’ for the Set A items, explained the voting process (see Appendix 7) and presented the items that would be considered for inclusion. Additional ‘*Recommended Reading’* explained how the CRG arrived at the Set A items and provided detailed item-specific information. The results of the Delphi study were presented numerically and graphically along with relevant free-text comments contributed by participants. Further relevant information derived from expert perspectives and other resources was also presented (e.g. related to sensitive, appropriate and inclusive wording of sex and gender items).

The eight items proposed for Set A were introduced and discussed separately and in turn, participants voted on their level of agreement with including each item. A detailed meeting report is provided in Appendix 8. In particular, there was extensive discussion related to the ‘race, ethnicity, culture and language’ item—including the challenge of defining globally-relevant and inclusive response categorisations, the varied use of terms and their varying acceptability, and legal issues in some countries. We pre-specified a threshold of 70% agreement for inclusion of items in Set A. Voting revealed high-level agreement for including seven of the eight items proposed for inclusion in Set A: at least 82% of participants either agreed or strongly agreed with their inclusion and contributed suggestions for minor changes to the wording and/or response options. While support to include the ‘race, ethnicity, culture and language’ item met our threshold for inclusion, only 48% agreed to include the item as it was proposed; 38% of participants agreed with including the item but had reservations (related to wording and response options), and 14% did not support including the item in Set A.

The lead author (ELK) summarised the discussion points from the transcribed audio-recording, collated the text-comments entered during the meeting and proposed changes to the items accordingly. These were reviewed and discussed by the CRG, leading to refinement of the wording and/or response options of all items and agreement on ‘preambles’ in line with meeting discussions. All modifications and additions were documented and forwarded to the Consensus Meeting attendees for further review.

Consensus Meeting #2: Two online meetings were conducted as step one of a two-step process to reach agreement on the Set B items. The first meeting was repeated to suit attendees across varied time zones and was recorded and shared with those unable to attend. The goal of the meeting was to provide preliminary information, explanation, and data to inform the completion of a follow-up online survey; and provide opportunity for questions/clarification. During the meeting, the facilitator (ELK) presented a summary of the results of the supplementary data survey—including the demographics of the participants involved and the item rankings. Consensus meeting participants were asked to consider these results alongside the findings of the Delphi study when making recommendations to include or exclude items. The content and format of the subsequent online survey was also explained. Twenty-five participants attended or viewed the online meeting and completed the survey, providing their opinion on including each of the 49 items being considered for Set B. Participant votes, their rationale for item inclusion/exclusion and suggestions for changes to the wording and/or response options were collated and reviewed by the CRG.

Nine items that were considered in the Delphi study but were ‘excluded’, were changed to ‘include’ based either on ratings of importance in the supplementary data survey, or provision of clear rationale to include them. Six items that were previously ‘included’ (based on Delphi results) were changed to ‘exclude’ (due to overlap/redundancy; or lack of specificity). Six of the items were combined to form a single item: “Have you been treated or judged unfairly due to any of the following reasons?”—with multiple response options. The wording and response categories were refined according to participant comments and suggestions, with further expert consultation undertaken where required. A total of 30 items, categorised according to PROGRESS-Plus, were finalised for inclusion in Set B and approved by all Consensus Meeting attendees.

#### STAGE 4: Focus groups

The focus group protocol was approved by the University of South Australia Ethics Committee (ID 205947) and pre-registered (https://osf.io/6zvpr/). Seven focus groups were conducted between August and November 2024 to explore the face validity and acceptability of the two ISSHOOs item sets. The groups involved participation from residents of all WHO Regions, including the Eastern Mediterranean Region.

One moderator (ELK) led two in-person ‘Researcher’ focus groups involving 19 pain researchers from 14 countries, in conjunction with the International Association for the Study of Pain World Congress in Amsterdam (Netherlands, August 2024). These focus groups were conducted in English and audio recorded. The recordings were later transcribed using Descript software^27^ and checked by ELK for accuracy. Five ‘Patient and Public’ focus groups were also conducted, involving a total of 20 participants from nine countries. Two groups occurred in-person with local group facilitators (in India and Chile), and three groups were conducted online, involving participants from Australia, Africa, and Europe. The discussion guides and a summary of participant characteristics are available in Appendices 9 and 10, respectively. Each of these focus groups were conducted and recorded using videoconferencing software (Zoom). The three online focus groups were conducted in English; the two in-person focus groups were conducted in participants native language (Marathi and Spanish)–lead by bilingual group facilitators who had received training from the lead investigator (ELK). The group facilitators interpreted the discussions in real-time and conveyed the translated information via the Zoom recording and additional notes (later provided to the lead investigator). All focus group comments were entered into Excel spreadsheets (organised by item). ELK identified themes and proposed suggestions for item refinements. The themes and suggestions were discussed with the CRG and item amendments were agreed upon by all group members.

A detailed summary of the main focus group discussion points and subsequent item modifications is provided in Appendix 11. Discussion of Set A led to minor modifications of six items, and major revision of item 5 (proposed to identify participants’ race, ethnicity and/or cultural background). Further development and refinement of Set A Item 5 occurred iteratively—involving feedback and discussion with focus group participants, relevant experts, and the author team. Discussion of Set B led to minor modifications to 21 of the 30 included items. The Researcher focus group discussions concluded by asking participants for their general perspectives on the ISSHOOs initiative. The following quotes capture the essence of focus group members perspectives:

“It seems like some populations, or some people are invisible, so we need to make them visible in order understand a little bit more about the disparities and if we don’t ask [these questions] then we can’t measure, and if we can’t measure, we can’t make hypotheses and if we don’t make hypotheses then things can’t change. So I think it’s really important.”

“I really appreciate this as a starting researcher ….I’ve been mulling through, creating my own set of these variables and it is really helpful to have something like this ready to use.”

#### STAGE 5: Final consultation, writing and dissemination

A final draft of the two item sets, with accompanying information necessary to guide implementation, was prepared by the CRG. Experts were consulted for further item-specific guidance where required, and the documents were circulated to the broader ‘ISSHOOs Group’ for final comments and approval. The current manuscript was prepared along with an ‘explanation and elaboration’ document, which is presented separately.^28^ In addition to the published manuscripts, the ISSHOOs datasets are available on the ENTRUST-PE website (https://entrust-pe.org/) and at https://www.isshoos.org/, where they are accompanied by further information and examples. Translated datasets and resources; and readily accessible data collection templates will be available.

### Role of the funding source

The funders had no role in the study design; in the collection, analysis, and interpretation of data; in the writing of the report; or in the decision to submit the article for publication.

## Results

The ISSHOOs recommendations comprise *ISSHOOs Set A:* eight items recommended for inclusion in all human adult pain research; and *ISSHOOs Set B:* 30 optional items for consideration. Both item-sets include a brief explanation of their purpose, and concise information (for researchers) to guide item tailoring and implementation. Additionally, we provide participant information and two item-specific preambles that are recommended to accompany data collection.

### ISSHOOs set A: the minimum dataset

#### 8 items recommended for inclusion in all human pain research involving adult participants

Set A is the recommended ‘minimum dataset’ comprising eight items: age; sex; gender identity; place of residence; race, ethnicity and/or cultural identity; education; financial position; and work status. Researchers are recommended to include all eight items in all pain-related studies involving human adult participants—to enable descriptions of study populations across a range of characteristics that are known to relate to health and health equity. Set A is detailed in full in Table 1; the items are accompanied by key information for researchers to facilitate understanding and implementation.

**Table 1:** The ISSHOOs (Identifying Social factors that Stratify Health Opportunities and Outcomes) ‘minimum dataset’: SET A.

### ISSHOOs set B: extended dataset

#### Optional equity-relevant items for pain researchers to consider including in human pain research involving adult participants

Set B (detailed in Table 2) comprises 30 optional items, categorised according to PROGRESS-Plus: Place (seven items); Race, ethnicity, culture and language (two items); Occupation (four items); Religion (two items); Education (two items); Socioeconomic position (three items); Social capital (five items); and ‘Plus’ (four items). Several items that crossed categories were combined to produce a single ‘discrimination’ item. The items included in Set B have been proposed and developed to collect data relating to participants' social, economic, and environmental circumstances. All of the Set B items were considered to be important determinants of health outcomes to the participants involved in the Delphi study. Researchers are recommended to consider including a subset of the items in Set B, as relevant to their research question(s), study setting(s) and participants. In particular, researchers are prompted to consider including the discrimination item (Item B1), due to its potential value for providing contextual understanding to some of the Set A item responses. Set B also includes brief information for researchers to guide item tailoring to suit specific study populations and contexts, and/or prompt consideration of additional ‘equity-relevant’ items not listed.

**Table 2:** The ISSHOOs (Identifying Social factors that Stratify Health Opportunities and Outcomes) ‘minimum dataset’: SET B.

## Discussion

The ISSHOOs datasets are the first consensus-derived recommendations for collecting and reporting a harmonised set of socio-demographic, equity-relevant data in all human adult pain research. These recommendations are the final outputs of a five-stage project that prioritised the participation of people with a lived experience of persistent pain, diverse and global engagement, and collaboration with interdisciplinary experts. The ISSHOOs items are potentially applicable to human adult health research well beyond the pain field.

The ISSHOOs recommendations fill an important gap by operationalising the collection of socio-demographic information across a comprehensive range of equity relevant characteristics, in a manner that has relevance and acceptability globally. Guidance to improve equity considerations in health research is widely available and addresses varied aspects of study methods and reporting. However, current initiatives commonly fall short of providing clear, practical guidance on what data to collect and how to collect it and are often limited in their scope. For example, in the pain field, the ‘Inclusion, Diversity, Equity, Antiracism, and Accessibility’ (IDEAA) guidelines prompt authors, reviewers, and editors to consider equity issues in study reporting.^29^ Their scope, however, does *not* include practical recommendations for collecting the relevant data to inform this reporting. Beyond the pain field, recommendations addressing the reporting of specific characteristics such as race and ethnicity,^8^^,^^30^ and sex and gender,^31^ exist; however, guidance documents that encompass a broad range of equity-relevant characteristics are scarce. Notably, the TrialForge initiative draws attention to a broad range of equity domains in its guidance for the handling of equity, diversity and inclusion in evidence syntheses, and provides a range of relevant resources to prompt diversity and inclusivity in clinical trials.^32^ The ISSHOOs recommendations are unique in their comprehensiveness, practical utility and global relevance; and will serve to valuably complement all of these initiatives.

Knowing what data to collect and how to collect it is challenging for researchers due to the complexity of issues, inconsistent policies, gaps in understanding, and a lack of clear guidance. ISSHOOs’ provides specific recommendations for questions and response categories that can be readily implemented. Where researchers are constrained by data-collection mandates from other sources (e.g. research funders)—they are encouraged to work within these limitations, consider congruence and where ISSHOOs may fill gaps. The information (for researchers and participants) that accompanies the ISSHOOs item-sets highlights the potential need for tailoring and will enable researchers to have confidence that they are collecting data related to the most important characteristics, in a manner that is sensitive, relevant and appropriate. Providing comprehensive guidance concerning the reporting of sociodemographic data is beyond the scope of this work: the acceptability of terms, categorisations and concepts can be highly varied, nuanced and complex; and diverse, context-dependent ethical frameworks exist. We encourage researchers to carefully consider cultural sensitivities, potential ethical issues, and the importance of maintaining participant anonymity. Guidance specifically addressing the reporting of sex and gender, and race and ethnicity in research are available (as noted previously); elaboration of these issues is also provided in the ISSHOOs Explanation and Elaboration manuscript.^28^

The ISSHOOs project meaningfully engaged people with lived experience, global experts (in methods, pain and health equity), and key interest holders throughout all project stages. This work is underpinned by research rigour with pre-registration and publication of study protocols, and transparent reporting of minor protocol deviations. Three manuscripts reporting major stages of this work have been published,^20^^,^^21^^,^^25^ in addition to a peer-reviewed correspondence manuscript providing project background and rationale.^15^

We made concerted and consistent efforts to engage people with diverse characteristics, particularly those who were geographically widespread or represented groups who are typically ‘hard-to-reach’. Our degree of success is both a strength and a limitation of this work. Our efforts to engage 55 ‘hard-to-reach’ participants subsequent to the Delphi study contributed data that importantly informed consensus meeting discussions and our final results. For example, participants from diverse cultural and religious groups (9 different religions) completed the Supplementary Data survey, allowing us to observe relationships between religious group membership and ratings of the importance of including items related to religion in the minimum dataset. Ratings were consistent with those in the Delphi study, providing reinforcement of the decision to *not* include an item related to religion in the minimum dataset. Researchers are guided, however, to select ‘religion’ items (or other items considered important) from Set B—as most relevant to their study participants, context or research questions.

Overall, we achieved significant global representation (45 out of 195 countries), but most countries were not represented. We acknowledge that there are many global perspectives that we have not heard in this work. While we incorporated native speakers of five of the six official WHO languages (excepting Russian), translated the surveys distributed in Stage 2 as required, and coordinated focus groups in Spanish and Marathi, the majority of contributors (overall) were native English speakers. Other ‘hard-to-reach’ groups also remained under-represented, for example people with low education and socio-economic positioning, people from rural and remote areas, and those who identify as belonging to minoritised or historically marginalised groups (e.g. based on cultural background, gender, or religious beliefs). We recognise that the outcomes of this project will be influenced by the characteristics and experiences of the participants involved; and that this has the potential to reduce the generalisability of our recommendations to other cultures, languages or socio-demographic groups. Researchers who aim to engage groups that have been under-represented in the development of these recommendations are encouraged to consider whether adaption of any of the ISSHOOs items is indicated. Community-engaged, participatory research processes (for example: Mittinty et al. (2022)^33^ and Lor et al. (2023)^34^ may be warranted to ensure that questionnaire items are relevant and acceptable in diverse study settings.

Concurrent with the publication of this manuscript, the ISSHOOs recommendations will be disseminated via national and international conference presentations and made available on the ISSHOOs website (https://www.isshoos.org/)—accompanied by additional resources including information, explanations, examples, translations and accessible files to assist implementation. Translations into the six official WHO languages will be developed and validated through a back-translation process (and made accessible on the website); machine-generated translations are provided in Appendices 12–16 in the interim. Detailed item-specific explanations, instructions and examples will be published in a separate ‘explanation and elaboration’ manuscript.^28^ The ISSHOOs Recommendations will be launched on Entrust-PE (https://entrust-pe.org/) (endorsed by the International Association for the Study of Pain), shared with the Cochrane Health Equity Thematic Group, and with funders of pain research, as a benchmark to guide standards for socio-demographic data collection and reporting. Crucially, we will request that pain journal editors endorse the outcomes of this project and consider our proposal to cross-publish a manuscript to raise awareness, understanding and implementation of the ISSHOOs outputs.

In future, systematic reviews of the reporting of equity-relevant data in published pain research will be conducted to provide indication of uptake and impact. We will endeavour to understand the acceptability and utility of the ISSHOOs recommendations; and seek feedback to inform refinements in line with evolving understanding and changing terminology. Potential next steps include considering population-specific extensions (e.g. for paediatric pain research; research involving indigenous groups or refugees; or research beyond the pain field) and developing automated tools or plug-in extensions for online survey software (e.g. REDCap). Future exploration of the potential for congruence with other data collection recommendations or mandates with overlapping objectives will also be worthwhile.

‘ISSHOOs’ offers pain researchers a *minimum dataset* of standardised, globally-applicable, equity-relevant items that can be readily implemented (Set A); and an *extended dataset* of optional items that researchers may consider using—relevant to their study population, setting and research questions (Set B). Through widespread uptake, the ISSHOOs recommendations will contribute to advancing health equity for people with pain, and they have potential for broader applications to other fields of health.

## Contributors

ELK and GLM conceived the original idea for the project and AGC, TB, MAB, AC, LJM, VM, JP, SS, PT made substantial contributions to conception and design. All members of the Core Research Group made substantial contributions to developing the protocol; and analysis and interpretation of data. AGC, AC and SS also contributed to the acquisition of data. All members of ‘The ISSHOOs Group’ made substantial contributions through their oversight, recruitment assistance, and participation in the Delphi study, Consensus Meetings and/or focus groups. ELK wrote the first draft of the manuscript. All authors have been involved in drafting the manuscript or revising it critically for intellectual content. They have all read and approved the final version. All members of the ISSHOOs Core Research Group (EKL, AGC, TB, MAB, AC, LJM, VM, JP, SS, PT and GLM) have accessed and verified all of the data and were responsible for the decision to submit the manuscript. The corresponding author attests that all listed authors meet authorship criteria and that no others meeting the criteria have been omitted.

## Data sharing statement

Data and materials are available from the corresponding author on reasonable request.

## Editor note

The Lancet Group takes a neutral position with respect to territorial claims in published maps and institutional affiliations.

## Declaration of interests

All authors have completed the ICMJE uniform disclosure form and declare:

Core Research Group:

ELK has received support from the National Health and Medical Research Council (NHMRC) Australia and is currently supported by The Mayday Fund. ELK has received speaker fees for lectures on pain and rehabilitation from professional and scientific bodies, and reimbursement of travel costs related to presentations at scientific conferences/symposia. ELK is an unpaid member of The International Association for the Study of Pain (IASP) Global Year 2025 on the theme of “Pain Management, Research, and Education in Low/Middle-Income Settings”, the IASP, and the Australian Pain Society.

AC is supported by grants from the European Horizon 2020 Research and Innovation Programme (Europe), the ZonMw Huisartsgeneeskunde en Ouderengeneeskunde (HGOG) Programme and the ZonMw Goed Gebruik Geneesmiddelen (GGG) Programme (The Netherlands).

SS was supported by the John J. Bonica Postdoctoral Fellowship from the IASP (2021–2023). The IASP did not have any influence on Dr Sharma's research. SS has received funding grant from the Medical Research Future Fund, the ANZ Back Pain Clinical Trials Networks and the National Health and Medical Research Council (NHMRC). SS has received financial support for travel costs related to presentations at scientific conferences, and renumeration for delivery of online lectures. SS has unpaid relationships with the following entities: SS is a board member for the Pain, Mind, and Movement Special Interest Group of the IASP, and the IASP Global Alliance for Partners in Pain Advocacy (GAPPA). SS is a trainee Editor for Pain Research Forum of the IASP and an Associate Editor for (1) the Journal of Orthopaedic & Sports Physical Therapy and (2) Physiotherapy. SS is Co-chair of The IASP Global Year 2025 on the theme of “Pain Management, Research, and Education in Low/Middle-Income Settings”. SS is a member of the IASP, Nepal Physiotherapy Association, and Australian Pain Society.

PT has received consulting fees to provide independent medical consultation and professional services. He is an independent Committee Member for clinical trial Data Safety Monitoring Boards for FDA approved trials being conducted by UCB Biopharma GmbH & SPRL, Parexel International, Prahealth Sciences. PT is an [unpaid] Chair of the Management Subcommittee of the Executive Committee of a registered non-profit independent medical research organisation, OMERACT.

GLM has received funding support from the NHMRC. GLM has received book royalties from books on pain and education from: NOIgroup publications; Dancing Giraffe Press; OPTP. GLM has received consulting fees from various sporting organisations; workers’ compensation boards; ConnectHealth UK; IOH California; Reality Health. He has received speaker fees for lectures on pain, pain education and rehabilitation from various professional societies; and travel and accommodation support for attendance at scientific meetings from Reality Health. GLM is an (unpaid) board member of the Australian Pain Solutions Alliance and has unpaid scientific advisor roles with the CRPS network and RSDSA.

The ISSHOOs Group:

OA is a voluntary member of The International Association for the Study of Pain (IASP) Global Year 2025 on the theme of “Pain Management, Research, and Education in Low/Middle-Income Settings”, a voluntary member of the Global Alliance of Partners for Pain Advocacy (GAPPA), Sickle Cell Disease Ambassador for Rare Disease South Africa, and Member of the Sickle Cell Disease Coalition (SCDC) for the American Society of Haematology (ASH).

DB has received payments and honoraria for lectures/presentations from Grunenthal.

MC has received grant support from the National Agency R&D Chile, and payments for lectures/presentations from Grunenthal Latin America. MC has unpaid roles on the IASP Council and as NeuPSIG Secretary.

MC (Cowern) has a paid leadership role as Head of Nations at UK Charity Versus Arthritis and an unpaid role as OMERACT PRP Support Team member.

BMF received support for attending European Pain Federation (EFIC) European Congresses while in her role as President of EFIC (2020–2023).

CH has received speaker payments from THINC Grant (CIHR) and received a stipend from OMERACT to attend bi-annual conferences. CH has a role in the Management Group at OMERACT.

FK has received grant support from MASS Pain Study in NC (2023–2024) and The BACk Study (2024-present). FK has received payments for delivering workshops and webinars associated with the NIH HEAL Initiative and has received travel support to attend national and international conferences/symposia from professional organisations (USASP, IASP, ACTTION, QPRN). FK held the role of treasurer of the International Network for Orofacial pain and Related disorders Methodology (INfORM) (2020–2023).

DL has received salary support from the HSS Dept of Anaesthesiology, Critical Care & Pain Management Research & Education Fund, and has received grant support from the NIH. DL has received travel support and payments for lectures/presentations from Analgesic, Anaesthetic, and Addiction Clinical Trials, Translations, Innovations and Opportunities Network (ACTTION).

BLT has received grant support from the Health Research Council, New Zealand; Medical Research Future Fund (Australia); and the Canadian Chiropractic Research Foundation. BL has received support to attend meetings at the University of Otago and holds a role in the Lived Experience Advisory Panel, NZ Pain Society. BLT writes the blog https://healthskills.wordpress.com and runs educational courses for health professionals in New Zealand.

JL has received annual payments from the Faculty for Honours Pain Course at University of Washington over the past decade.

TP has received grant/contract funding from the NIH; has received royalties from Oxford University Press and consulting fees from TriveniBio. TP is Editor-in-Chief, Journal of Pain.

RP has received grants to attend and participate in international research meetings from National Research Foundation of South Africa Knowledge, Interchange and Collaboration Grants (KIC23032487234; KIC240314209107; KIC24082824087). RP has received payment for lecturing on pain and pain management from Train Pain Academy not for profit organisation (South Africa), and payment for a webinar on pain from Heleon South Africa. RP is a councillor and fiduciary officer of IASP.

ASCR has received grants from UKRI (Medical Research Council & BBSRC), Versus Arthritis, Alan and Sheila Diamond Trust, Royal British Legion, European Commission, Ministry of Defence, Dr Jennie Gwynn Bequests, The British Pain Society, Royal Society of Medicine. ASCR undertakes consultancy and advisory board work for Imperial College Consultants–in the last 24 months this has included remunerated work for: AstraZeneca, Pharmnovo, Confo and Combigene. ASCR has received payments or honoraria for lectures/presentations from Hospital for Special Surgery/Cornell University, USA; and United Arab Emirates University. ASCR has a patent issued or pending for Rice A.S.C., Vandevoorde S. and Lambert D.M Methods using N-(2-propenyl) hexadecanamide and related amides to relieve pain. WO 2005/079 771. ASCR is President of IASP and has had roles on the following committees: Joint Committee on Vaccine and Immunisation-varicella sub-committee (until 2024); Medicines and Healthcare products Regulatory Agency (MHRA), Commission on Human Medicines–Neurology, Pain & Psychiatry Expert Advisory Group (until 2024); Analgesic Clinical Trial Translation: Innovations, Opportunities, and Networks (ACTTION) steering committee member (until 2024); Non-Freezing Cold Injury (NFCI) Independent Senior Advisory Committee (NISAC) (current).

ST is a member of the Board of Directors of the Australian Pain Society.

RDT has received grants from the European Union GA 777 500, DFG SFB 1158, GRK2350, IRTG 1874; and cash contributions to EU grant: Esteve, TEVA. RDT has received consulting fees from Bayer, Cered, Grünenthal, GSK, Merz, Sanofi; and support for meeting attendance, presentations and travel from several scientific societies. RDT has leadership or fiduciary roles in AWMF, IASP, EFIC, DGSS, WHO-MSAC.

JT has received payments and support to attend University of Ottawa Equity meetings and participate in STROBE research activities and manuscript writing.

AY is Councillor of the Pain in Childhood Special Interest Group, IASP; and Vice President of the Pain Association of Singapore.

There are no other relationships or activities that could appear to have influenced the submitted work.
