# Supplementary material for: Identifying Social factors that Stratify Health Opportunities and Outcomes (ISSHOOs) in pain research: consensus recommendations for the collection and reporting of equity-relevant data
Source: eClinicalMedicine. 2025 Oct 24;90:103586. doi: 10.1016/j.eclinm.2025.103586 (PMC12595279; doi:10.1016/j.eclinm.2025.103586)
Supplement: Appendix [file mmc1.docx]

**Supplementary File**

**Supplementary materials, containing Appendices 1 – 17.**

Page

Appendix 1: Alignment of the ISSHOOs Project with recommendations for the development of a reporting guideline …………………………………………………………………… 2

Appendix 2: Explanation of deviations from the original protocol …………………………………….. 3

Appendix 3: Interest-holder and Advisory Group members ……………………………………………. 4

Appendix 4: Initial list of candidate items for the Delphi study derived from the scoping reviews …… 5

Appendix 5: Stage 2: Summary of participant characteristics .… ……………………………………… 11

Appendix 6: Consensus meeting attendees ……………………………………………………………... 12

Appendix 7: Decision rules used to guide the consensus meetings …………………………………….. 14

Appendix 8: Summary of consensus meeting #1 ……………………………………………………….. 15

Appendix 9: Focus group discussion guide …………………………………………………………….. 23

Appendix 10: Focus group participant characteristics …………………………………………………… 24

Appendix 11: Focus group summary of results ………………………………………………………….. 26

Appendix 12: ISSHOOs Set A – Arabic ………………………………………………………………….. 29

Appendix 13: ISSHOOs Set A – Chinese ………………………………………………………………… 33

Appendix 14: ISSHOOs Set A – French …………………………………………………………………. 37

Appendix 15: ISSHOOs Set A – Russian ………………………………………………………………… 41

Appendix 16: ISSHOOs Set A – Spanish ………………………………………………………………… 45

**Appendix 1:** **Alignment of the ISSHOOs Project with recommendations for the development of a reporting guideline**

| Checklist of Steps (Moher et al. 2010) | ISSHOOs Project Activities |
| --- | --- |
| Initial Steps | |
| 1. Identify the need for a guideline | The need to develop new guidance was identified. The Core Research Group (CRG) was formed, and an ‘Interest and Advisory Group’ (IAG) subsequently established. |
| 1. Review the literature | Two Scoping Reviews of the literature carried out to identify current approaches to evaluating social determinants of health in health research and in clinical populations. |
| 1. Obtain funding for the initiative | N/a |
| Pre-(Consensus) Meeting Activities | |
| 1. Identify participants | We identified the global networks of the CRG and IAG and worked to expand these networks in order to achieve diverse, global representatives in all aspects of this project. |
| 1. Conduct a Delphi exercise | We conducted a 3-Round Delphi Study |
| 1. Generate a list of items for consideration at the Consensus Meeting | A list of items for consideration in the Consensus Meeting was generated from the results of (i) the Delphi Study (overall and disaggregated), and (ii) the Supplementary Data survey. |
| 1. Prepare for the Consensus Meeting | We decided on the size, duration and timing of the face-to-face meeting and required expertise. We developed the meeting agenda, meeting logistics, and allocated roles (timekeeper, facilitator etc). We prepared presentations of relevant background, a summary of data/results, and essential pre-meeting information (sent to participants prior to meeting). |
| The (Online) Face-to-Face Consensus Meeting | |
| 1. Present and discuss results of premeeting activities and relevant evidence | We presented all relevant data and information and the essential criteria for item inclusion. Participants voted on their agreement with including the proposed items and provided feedback and suggestions relating to item acceptability, global applicability, items wording and the range of response options. |
| Post-Meeting Activities | |
| 1. Develop the guidance statement (including pilot testing) | We conducted Focus Groups with Researchers (2 groups, total n=19) and ‘Patients and Public’ (5 groups, n=20 ). Data from the focus group discussions were used to refine the items and associated item pre-ambles. |
| 1. Develop an explanatory document | An explanation and elaboration document is being prepared and will be published separately. |
| 1. Develop a publication strategy | We discussed journal suitability (general medical vs pain-field-specific) and the potential for cross-publications. |
| Post-Publication Activities | |
| Steps 12 – 18. | (To follow). Will include encouragement of endorsement, support for adherence, development of website, translation of item sets, evaluation of impact, updating of resources. |

**Appendix 2: Explanation of deviations from the original protocol**

| Research stage | Explanation of protocol modification/addition |
| --- | --- |
| Delphi study: Round 3 | 1. We originally planned to include only the items for which agreement had not been reached (i.e. the ‘uncertain’ items) and ask participants to vote to ‘include’ or ‘exclude’ the items.   Variation: We included *all* of the items that had reached agreement to ‘include’ and asked participants to select up to 3 items (from each PROGRESS-Plus category) that they considered to be most important. We also asked participants to rate the importance of including 1 or more items from each PROGRESS-Plus category.  Rationale: We sought participant input to prioritise the ‘most important’ items from the included item set due to the large number of items that reached consensus to ‘include’.   1. We originally planned to ask for participant suggestions for refining the wording or response options of the items.   Variation: We did not seek this information during round 3 and instead planned to seek input into item refinements in the Consensus Meetings.  Rationale: This modification was made due to the large number of items the were included in Delphi round 3 and our focus on prioritisation of the items. |
| Supplementary data survey | We planned and conducted this additional project stage after realising that there were a number of groups that were under-represented in the Delphi study and that this presented a significant limitation to our findings. We targeted the recruitment of (typically) ‘hard to reach’ participants, who we asked to complete a modified version of the Delphi round 3 survey. |
| Consensus Meetings: structure | We initially planned a single Consensus Meeting.  Variation: We conducted a consensus process that involved two successive Consensus Meetings. The second meeting was conducted on 2 occasions to suit varied time zones.  Rationale: Following our decision to produce 2 datasets with some clear distinctions, it appeared most sensible, practical and feasible to run a 2-stage process to reach consensus on the Set A and Set B items (separately). |
| Focus groups:  Participants | We initially planned to conduct Focus Groups involving patient and public representatives (only).  Variation: We conducted 2 researcher-focus groups (in addition to the ‘patient and public’ focus groups.  Rationale: We considered it important to test the ISSHOOs datasets amongst the people who would be administering the questionnaires and took the opportunity to engage with the large global network of researchers attending the International Association for the Study of Pain World Congress to assemble 2 international focus groups. |
| Focus groups: Data analysis | In our original protocol we planned to analyse the focus group data using NVivo software.  Variation: ELK transcribed the focus group discussion and entered all data into Excel spreadsheets, organised according to PROGRESS-Plus and sub-categorised according to the item being discussed.  Rationale: Presenting the data in excel spreadsheets had benefits for organising the data, facilitating inductive content analysis (as planned) and clearly identifying and presenting the data to research colleagues for discussion. |

**Appendix 3: Interest-holder and Advisory Group members**

| Member | Country | Affiliation |
| --- | --- | --- |
| Oluwafemi Ajayi | Nigeria | Patient Advocate, Gail Sickle Initiative, University of South Africa |
| Cheryl Barnabe | Canada | Department of Medicine, McCaig Institute for Bone and Joint Health, University of Calgary |
| Monika Boogs | Australia | Pain Australia |
| Didier Bouhassira | France | Inserm U987, UVSQ, Paris-Saclay University, CHU Ambroise Pare, 92100 Boulogne-Billancourt, France |
| Margareta Calvo | Chile | Faculty of Biological Science, Faculty of Medicine, Pontificia Universidad Católica de Chile. |
| Mary Cowern | United Kingdom | Patient Research Partner, OMERACT |
| Karen Davis | Canada | Krembil Brain Institute, University Health Network; University of Toronto; Editor in Chief, PAIN |
| Maarten de Wit | Netherlands | Patient Partner |
| Brona Fullen | Ireland | UCD School of Public Health, Physiotherapy and Sports Science, Dublin Ireland. UCD Centre for Translational Pain Research |
| Catherine Hofstetter | Canada | Patient Research Partner, OMERACT |
| Mary Janevic (rep. USASP) | USA | Department of Health Behavior and Health Equity, University of Michigan School of Public Health, Ann Arbor, Michigan |
| Dale Langford | USA | Pain Prevention Research Center, Hospital for Special Surgery; Department of Anesthesiology, Weill Cornell Medicine |
| Bronwyn Lennox Thompson | New Zealand | Orthopaedic Surgery & Musculoskeletal Medicine, University of Otago, Christchurch |
| John Loeser | USA | Departments of Neurological Surgery and Anesthesia and Pain Medicine, University of Washington, Seattle, WA |
| Tonya Palermo | USA | Center for Child Health, Behavior and Development, Seattle Children’s Research Institute; Department of Anesthesiology & Pain Medicine, University of Washington |
| Mark Pitcher  (rep. Helene Langevin) | USA | National Center for Complementary and Integrative Health (NCCIH) at the National Institutes of Health (NIH), Maryland |
| Andrew Rice | United Kingdom | Pain Research Group, Dept Surgery and Cancer, Faculty of Medicine, Imperial College, London, UK. |
| Grayson Shultz | USA | Patient Advocate |
| Rolf-Detlef Treede | Germany | Department of neurophysiology MCTN, Department of Psychiatry and Psychotherapy CIMH, Heidelberg University, Mannheim |
| Janice Tufte | USA | PCORI Ambassador, Hassanah Consulting, Patient Partner |
| Angela Yeo | Singapore | Department of Paediatric Anaesthesia, KK Women’s and Children’s Hospital |

**Appendix 4: Initial list of candidate items for the Delphi study derived from the scoping reviews**

| Place of residence | |
| --- | --- |
| *Region of residence*   1. How would you describe where you live? | - Major city - Regional city - Large regional town - Small regional town - Remote area |
| *Living situation*   1. Which best describes your current living situation? | - I own a home - I rent a home - I live in my family’s home - I don’t have a place of my own and I have a place to stay - I don’t have a regular place to stay (e.g. homeless) |
| *Neighbourhood environment*   1. How easy is it for you to get to open-space areas with trees or other natural vegetation? | - Difficult - Somewhat difficult - Somewhat easy - Easy |
| *Neighbourhood safety*   1. Do you feel safe in your neighbourhood? | - Never - Rarely - Sometimes - Often - Always |
| *Access to transport*   1. Do you have access to reliable transportation? | - Never - Rarely - Sometimes - Often - Always |
| *Access to health services*   1. How easy is it for you to access the health care services you need? | - Easy - Somewhat easy - Somewhat difficult - Difficult |
| *Liveability*   1. How satisfied are you with the ‘livability’ of your neighbourhood? | - Very satisfied - Satisfied - Somewhat - Unsatisfied - Very unsatisfied |
| *Living conditions*   1. Do you have access to safe and reliable basic amenities? (E.g. water, electricity, toilet facilities) | - Never - Rarely - Sometimes - Often - Always |
| *Housing conditions*   1. How satisfied are you with the conditions in which you live? | - Very satisfied - Satisfied - Somewhat - Unsatisfied - Very unsatisfied |
| Race, ethnicity, culture and language | |
| *Country of birth*   1. What is your country of birth? | __ |
| *Country of residence*   1. Do you live in the country in which you were born? | - Yes - No |
| *Language*   1. Is the main language you speak at home different to the main language spoken where you live? | - Yes - No |
| *Ethnic origins or ancestry*   1. What are your ethnic origins or ancestry? Please select ALL the geographic areas from which your family’s ancestors first originated: | - Western Europe (e.g. Greece, Sweden….) - Eastern Europe (e.g. Hungary, Poland, Russia) - North Africa (e.g. Egypt, Morocco, Sudan) - Sub-Saharan Africa (e.g. Kenya, Nigeria…) - West Asia / Middle East (e.g. Iran, Israel…) - South and Southeast Asia (e.g. India…) - East and Central Asia (e.g. China, Japan…) - Pacific / Oceania (e.g. Australia, Fiji…) - North America (Canada, United States…) - Central America and Caribbean (e.g. Mexico) - South America (e.g. Brazil, Chile, Colombia) - Self-describe* [open text box] - Prefer not to disclose |
| *Racial background*   1. How would you identify yourself in terms of race? Please select ALL the groups that apply to you: | - Asian or Pacific Islander - Black - Hispanic or Latino/a/x - Indigenous (e.g. North American Indian Navajo, Aboriginal …) - Middle Eastern or North African - White - Self-describe* [open text box] - Prefer not to disclose |
| *Discrimination*   1. Have you felt treated or judged unfairly because of your racial or ethnic identity? | - Never - Rarely - Sometimes - Often - Always |
| *Country of residence*   1. How many years have you lived in the country where you now live? | - Less than 2 years - 2-5 years - 5-10 years - 10-20 years - More than 20 years |
| *Healthcare impacts*   1. Have you been treated or judged unfairly in a healthcare setting because of your racial or ethnic identity? | - Often - Sometimes - Rarely - Never - Not applicable |
| *Language*   1. Are you able to communicate easily (speak and write) in a language common to your region? | - Yes - No |
| *Language*   1. Do you fluently speak the main language spoken where you live? | - Yes - No |
| Minoritised/marginalised group   1. Does your race/ethnicity/culture/language represent a minority race/ethnicity/culture/language where you live? | - Yes - No |
| Occupation | |
| *Employment status*   1. What best describes your current work situation? | - Unemployed - Under-employed - Part-time or temporary work - Full-time work - Otherwise unemployed but not seeking work - Other (please specify): ___ - I choose not to answer this question |
| *Employment status*   1. Check…? | - Yes - No |
| *Occupation type*   1. Are you unemployed or without regular income? | ___________ |
| *Occupation type*   1. What term best describes your current occupation? | - Student - Blue collar worker* - White collar worker* - Self-employed - Retailer - Manager - Housekeeper - Unemployed - Retired |
| *Job satisfaction*   1. How satisfied are you with your current work situation? (Including job satisfaction, income, job security) | - Very satisfied - Satisfied - Somewhat - Unsatisfied - Very unsatisfied |
| *Occupational risks*   1. Does your work involve moderate or high risks? (E.g. of physical injury, toxic exposure, or well-being) | - Yes - No |
| *Health benefits*   1. Does your job include access to health benefits including insurance and/or compensation schemes? | - Yes - No |
| Gender and sex | |
| *Sex*   1. What is your sex? | - Male - Female - Another term |
| *Sex*   1. What sex were you assigned at birth? | - Female - Male - Intersex - Prefer to self-describe - Prefer not to answer |
| *Gender*   1. How do you describe your gender? | - Man or male - Woman or Female - Non-binary - I use a different term (please specify: ____ ) - Prefer not to answer |
| *Gender identity*   1. With which gender do you most identify? | - Man or male - Woman or Female - Non-binary - I use a different term (please specify: ____ ) - Prefer not to answer |
| *Discrimination*   1. Have you been discriminated against because of your sex or gender identity? | - Often - Sometimes - Rarely - Never |
| Religion | |
| *Religious belief*   1. What is your religion? | - No religion - Christian - Islam - Buddhism - Hinduism - Judaism - Sikhism - Other (please specify:____) |
| *Religiosity*   1. Are you an active member of a religious community? | - Yes - Somewhat - No |
| *Discrimination*   1. Have you felt treated or judged unfairly because of your religion? | - Never - Rarely - Sometimes - Often - Always - Not Applicable |
| *Impact on healthcare*   1. Have your religious beliefs negatively impacted your pain care? | - Yes - Somewhat - No |
| Education | |
| *Years of education*   1. How many years of education have you completed? | __ years |
| *Educational attainment*   1. Which of the following best describes your highest level of education? | - No formal schooling - Primary school - Secondary school - Vocational or trade school - 2-4 year College/Associate/Bachelor's degree - Master's degree - Doctoral degree - Other (please specify): ___ |
| *Maternal/ Paternal education*   1. How would you best describe the education status of your mother (/father)? | - Illiterate - Read and write - Primary - Secondary - College or above |
| *Health literacy*   1. How confident are you filling out medical forms by yourself? | - Extremely - Quite a bit - Somewhat - A little bit - Not at all |
| Socioeconomic status | |
| *Financial stability*   1. How well are you able to meet your basic living expenses? | - Just Meet - Meet Adequately - Meet Comfortably - Cannot meet |
| *Financial stability*   1. How would you say you are managing financially at the moment? | - Living comfortably - Getting by - Finding it difficult |
| *Financial stability*   1. Over the past month, did you and your family have a stable income? | - Yes - Sometimes - No |
| *Financial stability*   1. Do you and your family have enough money for the things you need (food, rent, medicines, bills)? | - Yes - Sometimes - No |
| *Household/ family income*   1. What is your average monthly household income (approximately)? | ___________ |
| *Healthcare affordability*   1. In the 6 months, have you needed to see a doctor or take medications but could not because it costs too much? | - Yes - No |
| *Postcode classification*   1. What is your postcode? | ___________ |
| *Financial support*   1. Do you receive any financial/basic needs support from the government or community programs? (e.g. food assistance, disability or child support grants) | - All of the time - Some of the time - Rarely - Never |
| *Health insurance*   1. Do you have affordable access to health insurance? | - Yes - No |
| *Affordability of pain care*   1. Do your finances limit your access to optimal pain care? | - Yes - Sometimes - No |
| Social capital | |
| *Marital/relationship status*   1. What is your relationship status? (Select all that apply) | - Single - Married - Living with a partner - In a relationship or engaged - Separated or divorced - Widowed - It’s hard to explain/prefer not to answer |
| *Co-habitants/living arrangements*   1. Which of the following best describes your living arrangements? | - Live with partner only - Live with partner and children - Sole parent with children - Live with parents/other related adults - Live with unrelated adults - Other (please specify): ___ |
| *Social connectedness*   1. During the past 12 months, how often have you felt lonely? | - Never - Rarely - Sometimes - Most of the time - Always |
| *Social connectedness*   1. How often do you see or talk to people that you care about and feel close to? (E.g. talking to friends, visiting friends/family, going to church or club meetings) | - Less than once a week - 1-2 days a week - 3-4 days a week - 5 or more days a week |
| *Connection to community*   1. How closely do you feel connected to the community in which you live? | - Not connected at all - Somewhat connected - Connected - Very connected |
| *Quality of relationships*   1. In general, how fulfilled are you in your relationships with others? | - Very fulfilled - Fulfilled - Somewhat fulfilled - Unfulfilled |
| *Social support*   1. Do you have someone you can rely on in case of need/emergency? | - Yes - No |
| Plus | |
| *Age*   1. How old are you? | ___ (years) |
| *Date of birth*   1. What is your date of birth? | day___ month ___ year___ |
| *Sexual orientation*   1. How do you describe your sexual orientation? | - Straight (heterosexual) - Gay or lesbian - Bisexual - I use a different term (please specify) - I don’t know - Prefer not to answer |
| *Sexual orientation/ discrimination*   1. Have you ever felt treated or judged unfairly because of your sexual orientation? | - Never - Rarely - Sometimes - Often - Always - Not Applicable - I’d rather not say |
| *Chronic disease/disability*   1. Have you ever felt treated or judged unfairly due to a long-term disability? | - Never - Rarely - Sometimes - Often - Always - Not Applicable - I’d rather not say |
| *Interpersonal violence or safety*   1. Do you and/or your children feel physically and emotionally safe in your home? | - Yes - No - Unsure - I choose not to answer this question |
| *Internet access and connectivity*   1. Over the past month, did you and your family have stable access to internet or mobile data? | - Yes - No |

**Appendix 5:** **Stage 2: Summary of participant characteristics**

| Participant characteristics | Delphi Study (n=168)*  n (%) | | Supplementary Data Survey (n=55)**  n(%) | | |
| --- | --- | --- | --- | --- | --- |
| Age (years) | | | | | |
| 18-24 | 3 (2) | | 7 (14) | | |
| 25-34 | 21 (13) | | 21 (40) | | |
| 35-44 | 59 (35) | | 9 (17) | | |
| 45-54 | 36 (21) | | 8 (15) | | |
| 55-64 | 23 (14) | | 5 (10) | | |
| 65-74 | 16 (10) | | 2 (4) | | |
| 75+ | 10 (6) | | - | | |
| Continent of residence | | | | | |
| Africa | 23 (14) | | 20 (39) | | |
| Asia | 29 (17) | | 27 (52) | | |
| Europe | 30 (18) | |  | | |
| North America | 45 (27) | | - | | |
| Oceania | 32 (19) | | 5 (10) | | |
| South America | 10 (6) | | - | | |
| Country of residence | | | | | |
|  | (see below)^a^ | | (see below)^b^ | | |
| Region of residence | | | | | |
| Capital/major city | 106 (63) | | City | 20 (38) | |
| Regional city | 28 (17) | | Town/suburb | 21 (40) | |
| Large regional town | 13 (8) | | Rural area | 11 (21) | |
| Small regional town | 19 (11) | |  |  | |
| Remote area | 2 (1) | |  |  | |
| Sex (assigned at birth) | | | | | |
| Female | 101 (60) | | 20 (38) | | |
| Male | 65 (39) | | 31 (60) | | |
| Intersex | - | | - | | |
| Prefer to self-describe | - | | - | | |
| Prefer not to answer | 2 (1) | | 1 (2) | | |
| Gender | | | | | |
| Woman/female | 99 (59) | | 22 (42) | | |
| Man/male | 66 (39) | | 25 (48) | | |
| Non-binary | 3 (2) | | 1 (2) | | |
| I use a different term | -^†^ | | - | | |
| I am not sure or questioning | -^†^ | | 1 (2) | | |
| I don’t know what the question means | -^†^ | | 2 () | | |
| Prefer not to answer | - | | - | | |
| Highest level of education | | | | | |
| No or limited formal schooling | - | | 6 (12) | | |
| Primary school | - | | 1 (2) | | |
| Lower secondary or middle school | - | | 7 (13) | | |
| Upper secondary or high school | 1 (1) | | 10 (19) | | |
| Further education, non-university | 5 (3) | | 8 (15) | | |
| Some college or university | -^†^ | | 6 (12) | | |
| Tertiary: Grad. Dip./Bachelors degree | 39 (23) | | 8 (15) | | |
| Tertiary: Masters degree | 39 (23) | | 5 (10) | | |
| Tertiary: Doctoral degree | 84 (50) | | (included in row above) | | |
| Language spoken at home | | | | | |
| English | 127 (76) | | 6 (12) | | |
| Languages other than English | 41 (24)^c^ | | 46^d^ | | |
| Financial status | | | | | |
| *How are you managing financially at the moment?* | | | *How easily can you meet your basic living expenses?* | | |
| Living comfortably | 114 (68) | | Can meet very easily | | 2 (4) |
| Getting by | 37 (22) | | Can meet easily | | 9 (17) |
| Finding it difficult | 14 (8) | | Can just meet | | 13 (25) |
| I’d rather not say | 3 (2) | | Can meet sometimes but not always | | 20 (38) |
|  |  | | Cannot meet | | 7 (13) |
|  |  | | Prefer not to answer | | 1 (2) |
| Ethnic origins or ancestry (more than one selection permitted) | | | | | |
| Western Europe | 88 (52) | | 7 (13) | | |
| Eastern Europe | 12 (7) | | - | | |
| North Africa | 3 (2) | | - | | |
| Sub-Saharan Africa | 17 (10) | | 20 (38) | | |
| West Asia / Middle East | 5 (3) | | 14 (27) | | |
| South and Southeast Asia | 34 (20) | | 8 (15) | | |
| East and Central Asia | 7 (4) | | 1 (2) | | |
| Pacific / Oceania | 7 (4) | | 2 (4) | | |
| North America | 14 (8) | | - | | |
| Central America and Caribbean | 10 (6) | | - | | |
| South America | 12 (7) | | - | | |
| Mixed/multiple backgrounds | -^†^ | | 1 (2) | | |
| Not sure/prefer to not disclose | 3 (2) | | - | | |
| Indigenous/First Nations | | | | | |
| Yes | 30 (18) | | 1 (2) | | |
| No | 131 (78) | | - | | |
| Prefer not to answer | 7 (4) | | - | | |
| Identify as belonging to a minoritised group based on race, ethnicity or cultural identity | | | | | |
| Yes | -^†^ | | 25 (48) | | |
| No | -^†^ | | 22 (42) | | |
| Prefer not to say | -^†^ | | 5 (10) | | |
| Religion | | | | | |
| No religion | | -^†^ | 10 (19) | | |
| Prefer not to answer | | -^†^ | 3 (6) | | |
| Self-reported religion | | -^†^ | (see below)^e^ | | |
| Sexual orientation | | | | | |
| Bisexual | | -^†^ | 2 (4) | | |
| Gay or lesbian | | -^†^ | 9 (17) | | |
| I don’t know | | -^†^ | 2 (4) | | |
| I use a different term | | -^†^ | 1 (2) | | |
| Pansexual | | -^†^ | 1 (2) | | |
| Prefer not to answer | | -^†^ | 2 (2) | | |
| Heterosexual | | -^†^ | 35 (67) | | |

*35% with a lived experience of persistent pain

**73% with a lived experience of persistent pain

^†^Question not asked

^a^United States of America (33), Australia (23), Canada (12), South Africa (12), New Zealand (9), United Kingdom (9), Bhutan (7), Ghana (6), Chile (4), Netherlands (4), Pakistan (4), Singapore (4), Brazil (3), Germany (3), Nigeria (3), Portugal (3); Bangladesh, Belgium, Colombia, Denmark, India, Ireland, Nepal, Philippines (2); Benin, Ethiopia, France, Indonesia, Iran, Iraq, Ireland, Lebanon, Mongolia, Norway, Paraguay, Sweden, Switzerland, Thailand (1)

^b^Australia (5), Bhutan (1), Cambodia (2), Ghana (20), India (4), Iran (1), Iraq (8), Saudi Arabia (6), Syrian Arab Republic (5)

^c^Afrikaans, Amharic, Arabic, Bangla, Bengali, Bulgarian, Cantonese, Danish, Dutch, Dzongkha, Ewe, Fante, Filipino, Flemish, French, German, Guarani, Hindi, Indonesian, Italian, Kannada, Kurdish, Mandarin, Mongolian, Nepali, Norwegian, Persian (Farsi), Portuguese, Punjabi, Sindhi, Sorani, Spanish, Swedish, Tamil, Telugu, Thai, Tibetan, Twi, Two, Urdu, Yoruba

^d^Arabic, Dagati, Dagbani, Dende, Dzongkha, Fante, Fullani, Gonja, Hausa, Hindi, Kannada, Khmer, Konkonba, Kurdish, Tshangla,Twi, Urdu

^e^Alawite (1), Buddhist (2), Christian (12), Hindu (2), Islam (18), Kurdish (1),Quaker (1), Reddy’s/Hundes (1), Traditionalist (1)

**Appendix 6: Consensus meeting attendees**

|  | Country | Expertise/affiliation |
| --- | --- | --- |
| Core Research Group (n=10) | | |
| Trevor Barker  Mark Boyd  Aidan Cashin  Alessandro Chiarotto  Emma Karran  Lara Maxwell  Vina Mohabir  Lorimer Moseley  Saurab Sharma  Peter Tugwell | Australia  Australia  Australia  The Netherlands  Australia  Canada  Canada  Australia  Australia/Nepal  Canada | Patient-partner  Chair of Medicine, ​University of Adelaide  Research Fellow, Neuroscience Research Australia & University of New South Wales  Assistant Professor, Department of General Practice, Erasmus MC  Postdoctoral Research Fellow, University of South Australia,  Research Coordinator Managing Editor Cochrane Musculoskeletal Group, University of Ottawa  Patient Partner, Clinical Research Project Coordinator, ​The Hospital for Sick Children  AO, Bradley Distinguished Professor, Professor of Clinical Neurosciences, ​Chair in Physiotherapy University of South Australia  Chief Clinical Scientist, Pain Management and Research Centre, Royal North Shore Hospital  OC, Professor & holder of the Canada Research Chair ​in Health Equity, The Ottawa Centre for Health Equity, ​University of Ottawa |
| Interest-holder and Advisory Group (n=15) | | |
| Oluwafemi Ajayi  Didier Bouhassira  Margarita Calvo  Karen Davis  Brona Fullen  Mary Janevic  Dale Langford  Bronnie Lennox-Thompson  John Loeser  Romy Parker  Mark Pitcher  Tonya Palermo  Andrew Rice  Grayson Shultz  Janice Tufte | Nigeria  France  Chile  Canada  Ireland  United States of America  United States of America  New Zealand  United States of America  South Africa  United States of America  United States of America  United Kingdom  United States of America  United States of America | Patient partner; founder of Gail Sickle Initiative; PhD Candidate, University of South Africa  Editor-in-Chief, European Journal of Pain; Director of Research (Inserm)  Ass. Prof. Pontificia Universidad Católica de Chile, International Association for the Study of Pain  Main Editor, PAIN, Prof. of Neuroscience, University of Toronto  Ass. Prof. University College Dublin, Past President European Pain Federation  Ass. Prof. University of Michigan, Elected representative United States Association for the Study of Pain  Ass. Prof; Director, Chronic Pain Research, Hospital for Special Surgery  Senior Lecturer, University of Otago, Occupational Therapist, lived experience  Prof Emeritus, Anaesthesiology & Pain Medicine, University of Washington  Ass. Prof. University of Cape Town & Groote Schuur Hospital  National Institutes of Health (NIH) representative  Editor-in-Chief, Journal of Pain, Prof. University of Washington  Prof. Pain Research, Imperial College London, President, International Association for the Study of Pain  Patient advocate, expertise in sexuality, gender, disability, chronic disease  Public representative and patient collaborator, PCORI Ambassador, Hassanah Consulting |
| Other invited representatives (N=7) | | |
| Ruth Appiah  Sónia Bernades  Robert Hurley  Flavia Kapos  Karma Phuentsho  Rafael Zambelli Pinto  Audrey Wang  Amanda C de C Williams | Ghana  Portugal  United States of America  Brazil, United States of America  Bhutan  Brazil  Malaysia, China, Australia  United Kingdom | Medicine, PhD Candidate, University of South Australia  Ass Prof (Psychology) Iscte-University Institute of Lisbon  Editor-in-Chief, Pain Medicine, Prof. Anaesthesiology, Wake Forest University  Ass. Prof. Orthopaedic Surgery (Dentistry) Duke University  Physiotherapist, PhD Candidate, University of South Australia  Editor-in-Chief, Brazilian Journal of Physiotherapy, Adjunct Prof. Universidade Federal de Minas Gerais  Senior Lecturer in Digital Health, University of Sydney  Prof. Clinical Health Psychology, Social Psychology University of Bristol |

**Appendix 7: Decision rules that were used to guide the consensus meetings**

Essential Criteria for the Items Included in Set A:

The items included in SET A *must:*

1. *Describe* the characteristics of the study participants (adults)
2. *Specifically* describe the characteristic of interest
3. Be *acceptable* cross-culturally
4. Be *relevant* to diverse study settings & global contexts
5. Be *implementable* across all study methods
6. Use *sensitive* & *inclusive* wording*
7. Be *concise, translatable & self-explanatory**

Voting:

*I support the inclusion of this item in SET A:*

*
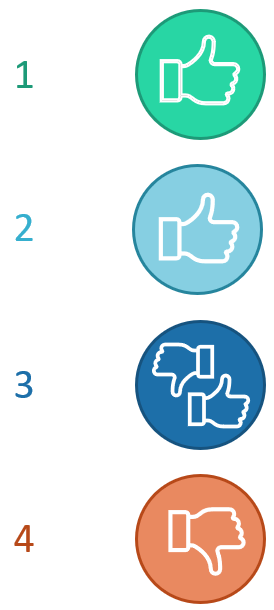
*

**Strongly agree**

*I support including this item*

- No changes are needed

**Agree**

*I support including this item*

- Changes to wording and/or response options need to be considered

**Reservations**

*I have some problems with including this item, but I can go along with it*

- Changes to wording and/or response options may also need to be considered

**Disagree**

*I have a fundamental disagreement & do not support including this item*

**Appendix 8: Summary of consensus meeting #1**

Important general guidance for researchers:

For all items consider:

1. *Tailoring* the language or response categories to suit the local study context/population
2. *Deleting* any response categories that are not relevant
3. The need for *additional items*. Researchers may need to collect further demographic data depending on their study context, participants, and research questions.

(Overall) pre-amble for study participants: *The following questions ask for some personal information. Your answers will be kept safe and reported as ‘group answers’ only. This means that no one can know it’s you. It is usual for researchers to ask some personal questions so that they can describe the people in their study. This information may also be used to understand more about the factors that influence health – with the aim of improving health outcomes for everyone.*

PLACE

| Main discussion points | Summary of ‘chat notes’ |
| --- | --- |
| - Suggest changing the question to: “Where do you live” - Consider using the world bank criteria of density to define the options.   Extensive discussion around response options – e.g:   - Inclusion of ‘village’ and where it fits best? (additional or substitute category) - City - suggest adding: ‘including surrounding suburbs/ surrounding metropolitan area’ | - In Canada, there is an index of remoteness <https://www150.statcan.gc.ca/n1/en/catalogue/172600012020001>. In Australia, Remoteness Areas divide Australia into 5 classes of remoteness on the basis of a measure of relative access to services <https://www.abs.gov.au/ausstats/abs@.nsf/mf/1270.0.55.005>. - Numerous comments related to number of response options/varied classifications: e.g. inclusion of village (substitute or additional category), remote/very remote - Consider evaluating density & distance (e.g. from health care services)? - Maybe we need to ask 2 questions: how far (time wise) do you live from a health centre, and how dense is the place where you live? - Need to first decide what it is that you want to understand with this question and then you can set up the question... for example if you want to know about access, then it is about distance but not always given how many people the health care system is serving... also, you may be wanting to know about access to speciality/high level care/technology. |
| *Proposed Item:*  How would you describe where you live?  o City  o Town or village  o Rural area  *Vote result:*  *Strongly agree – 3%*  *Agree – 83%*  *Reservations – 13%*  *Disagree – 0%* | *Refined Item:*  Notes for researchers:   - The response options for this item are consistent with the World Bank definitions defining [Degree of Urbanisation](https://blogs.worldbank.org/sustainablecities/how-do-we-define-cities-towns-and-rural-areas%20.) - Information and guidance will accompany this item – so that researchers understand the 3-level classification and are able to modify the wording of the response options to suit the local context.   Variable name: Place of residence  *Item:* Where do you live?   - City - Town - Rural area - Prefer not to answer |

RACE, ETHNICITY, CULTURE & LANGUAGE

| Main discussion points | Summary of ‘chat notes’ |
| --- | --- |
| - Definition of Hispanic/Latino is vague.... and interpreted differently in different countries - This category is very troublesome! - These categorisations should be derived in the specific context and with the agreement of the people in the communities. - From a European perspective these categorisations do not apply. In France I cannot ask my patients about their race. Race is not recognised in France, Germany and in many European countries. This classification is completely illegal – if I ask this of my patients I could be sued. Even ethnicity is extremely sensitive and not well defined. Sociological definition of race varies across countries. - Use of terms race and ethnicity are problematic. We need to put this question as optional. - Need a pre-amble to explain why we are asking this question. I am very glad it is asked – it shows up inequities in health that we otherwise wouldn’t be able to see. - Look at the American Psychological Association classification on race and ethnicity. Suggest how to tailor it to specific culture and communities. - Ask ourselves: why do we want to know this information and what are we trying to get – then we need to say that in a pre-amble. - I would certainly take out the word race and probably ethnicity. | - Indigenous is the term most often used in Canada but there are complexities … would never use terms like "Indian" or "aboriginal" or "eskimo" - Comments regarding wording: Hispanic/Non-Hispanic, Latino…. - For this item would a mixed race South African, who call themselves Coloured and who have a distinct culture be included in Self-describe? Or would local tailoring allow for that? - In the US Pacific Islanders really want to be recognized separate than Asian - NZ has requirements for reporting ethnicity - it's important to let researchers know that items can be tailored - The options for this question will most likely be tailored for each geographic location - Allow self-description - In the US, "Middle Eastern or North African" is a common category. I don't think North African is on the current list. - Race and ethnicity are distinct (albeit usually related) constructs. There is a lot of mixing of those constructs in these response options. - It may be good to remove 'European descent' from white, simply because that could be taken to suggest that being of European descent is always related to whiteness. - Our German and French colleagues are unable to collect these data - Are we trying to understand the social factors or the biology... I would argue you may never to know the biology due to generations of intermarriage, movement, etc. - It’s critical to ensure our research works hard to include people who are marginalised because of ethnicity - Skin colour is problematic in describing ethnicity. We should definitely not include Another race/ ethnic group? It should say Other ethnic you identify with? - I think the reason to include this item is to ensure, e.g. in the context of a clinical trial, that the findings are generalizable to racially & ethnically diverse populations - Could refer to the American Psychological Association 2019 Guidelines on Race and Ethnicity in Psychology? - Culture/Language can be sometimes different from ethnicity. “What culture, ethnicity and language do you identify with?” might be more inclusive. - Classification needs to be done in a way that allows grouping later on |
| *Proposed item:*  Which category best describes how you identify? (Select all that apply)   - Black (e.g., African, Afro-Canadian, Afro-Caribbean, Afro-Egyptian etc.) - East Asian (e.g., Chinese, Korean, Japanese, Taiwanese, etc.) - Middle Eastern, Arab or West Asian (e.g., Afghan, Egyptian, Iranian, Lebanese, Persian, Turkish, Kurdish, etc.) - Hispanic or Latino/a/e/x - Indigenous (e.g. American Indian, Alaska Native, First Nations, First Peoples, Aboriginal, Torres Strait Islander, Māori, Native Hawaiian or Other Pacific Islander) - South Asian (e.g., Bangladeshi, Indian, Indo-Caribbean, Pakistani, Sri Lankan, etc.) - Southeast Asian (e.g., Filipino, Vietnamese, Cambodian, Thai, Indonesian, etc.) - White (e.g. European descent) - Another race/ethnic group (please specify: ­­­­­________) - Do not know - Prefer not to answer   *Vote result:*  *Strongly agree – 7%*  *Agree – 41%*  *Reservations – 38%*  *Disagree – 14%* | *Refined Item:*  Notes for researchers:   - The response options listed in the examples below are unlikely to cover every possible identity. - It is important to be sensitive to the cultural nuances and diverse identities that exist worldwide. Depending on the context of your study and the specific population you may need to tailor the response options to ensure inclusivity. - Where possible, use classifications that have been derived in the country in which the study is being conducted and are considered acceptable by the local population. - Researchers should remove this item if they are concerned it will cause offence or contradicts local laws or customs - Two examples are provided (i) an item that could be considered for use in a global study; (ii) an item that has been tailored for an Italian population.   Variable name: Ethnicity and/or culture  Item: Which category best describes how you identify? (Select all that apply)   - Northern African - Sub-Saharan African - Latin American or Caribbean - Northern American - Central Asian - Eastern Asian - South-Eastern Asian - Southern Asian - Western Asian - Eastern European - Northern European - Southern European - Western European - Australian or New Zealander - Melanesian or Micronesian or Polynesian - Black - Hispanic or Latino/a/x - Indigenous - White - Mixed/multiple backgrounds - Another category (please specify: ______) - Prefer not to answer |

OCCUPATION

| Main discussion points | Summary of ‘chat notes’ |
| --- | --- |
| - Suggest adding ‘student’ to the options - Prefer ‘not working’ to unemployed. Otherwise culturally appropriate - Some additional discussion around categorisations | - Additional categories to consider: ‘student’, ‘self-employed?’ ‘on leave (parental, medical, study etc), ‘unable to work’ - Need an explanation for the item 'insecure work’? - Consider alternatives to "homemaker" - Need to clarify unemployed options - Suggest separating retired and disability pension into separate options - Underemployment is a problem we are not identifying. Could be difficult to understand and/or translate. 'Casual work' is not a common notion in Ghana - Clarify the purpose of this question - Use “Work Status” rather than “Occupation” to describe this item - Part time worker, full time worker (rather than work) - Can we split casual, temporary from insecure? Casual and temporary might be a choice. Insecure work to me implies its forced - Try to condense the set of response options to avoid it being too lengthy |
| *Proposed item:*  What best describes your current work situation? (Select all that apply)  o Unemployed  o Seeking work  o Casual, temporary or insecure work  o Part-time work  o Full-time work  o Homemaker, 'at-home' parent or (unpaid) carer  o Unemployed but not seeking work (e.g. retired,  disability pension)  o Other (please specify: ____)  o Prefer not to answer  *Vote result:*  *Strongly agree – 4%*  *Agree – 89%*  *Reservations – 7%*  *Disagree - 0* | *Refined Item:*  Variable name: Working status  Item: What best describes your current work situation? *(Select all that apply)*   - Part-time worker - Full-time worker - Not working   Select any reasons for not working you would like to provide (you can select more than one):   - - On disability or medical leave   - ‘At home' parent/home manager/caregiver   - Student   - Retired - Other (please specify: ______) - Prefer not to answer |

GENDER & SEX

| Main discussion points | Summary of ‘chat notes’ |
| --- | --- |
| - A pre-amble is important - Gender identity: using man or male is very confusing – these are not interchangeable terms. I would take out the male and female terms. If the non-binary term is taken out it might be less sensitive (people can use ‘another term’) and more globally applicable. - Many cis gender people do not understand the difference between male and man and so if they do not see ‘male’ they might click ‘another term’ so that they can insert male. Because it is politicised it is helpful that they are both listed here. - Culturally acceptable in Bhutan. Agree that cisgender may get confused with some of the terms - In Ghana it is illegal to be anything other than male/female - Consider translations: to say “man or male” would be very difficult – not translatable.(France) | - A pre-amble is important to explain what we’re asking and why. Consider describing what "assigned at birth" means. - Comments regarding use of man/woman and male/female terms - I think researchers misuse and misunderstand the terms sex and gender - It's important to note this isn't about sexuality so needs a preamble so responders can make sense of the question - In some of our languages we may not have language for all the gender options. Non-binary in particular. - Consider 'third gender' – but this would be confusing in some cultural contexts - I would vote to remove the response option "I don't know what this question means" - might serve to further minoritize individuals who are gender diverse - The already listed options currently capture the diverse genders from my perspective and are quite clear. |
| *Proposed item:*  What sex were you assigned at birth?  o Female  o Male  o Another term (please specify: _______)  o Prefer not to answer  *Vote result:*  *Strongly agree – 71%*  *Agree – 25%*  *Reservations – 4%*  *Disagree - 0*  Which of the following terms best describes your current gender identity?  o Man or male  o Woman or Female  o Non-binary  o Another term (please specify: _______)  o I am not sure or questioning  o I don't know what this question means  o Prefer not to answer  *Vote result:*  *Strongly agree – 10%*  *Agree – 72%*  *Reservations – 17%*  *Disagree - 0* | *Refined Item:*  Notes for researchers:   - We recommend providing a preamble with the following items (as detailed below) - In some study settings it may be most appropriate to *not* include the preamble and modify the response options due to political, cultural, religious, safety, or other reasons   Variable name: Sex  Preamble: *Sex is about the biological parts of a person. This includes things like chromosomes, genes, hormones, and body parts related to reproduction and sex. We often think of sex as being either female or male, but there can be other variations.*  Item: What sex were you assigned at birth?   - Female - Male - Another term (please specify: _______) - Prefer not to answer   Variable name: Gender  Preamble: *Gender identity is how someone feels about their own gender. There are many ways a person can describe their gender identity and many labels a person can use*.  Item: Which of the following terms best describes your current gender identity?   - Man - Woman - Non-binary - Another term (please specify: _______) - I am not sure or questioning - I don't know what this question means - Prefer not to answer |

EDUCATION

| Main discussion points | Summary of ‘chat notes’ |
| --- | --- |
| - Educational status: in Latin American countries, most people now finish primary school but do not complete secondary school but is very different to have someone only completing primary school versus completing 3 years of secondary, and that will give you the same result. Also, primary/elementary, and secondary/high school vary a lot depending on the country (in Chile primary is 9 years, in the UK is 6-7 years, etc). I think it would be better to ask for the number of years done at school and the number of years done at university. - A couple of categories missing. Differentiating GED from a high school diploma, and ‘some college’ (rather than requiring completion). - Vocational/trade school might exclude apprenticeships – the “school” term is the bit that doesn’t fit - Do we need a special option for Professional Degrees, e.g. MDs, JDs – probably need to be a specific as we can with peoples educational background – it allows us to do different things in life. - Are you looking at someone’s education or are you looking at someone’s ability to practice a trade. I think you’ve mixed them here. - Is this question also about health literacy and if it is has your group considered talking to health literacy people? Also consider: In different countries vocational trade pays really well and in other countries it doesn’t. | - ‘Some college’ and ‘some graduate school ‘ is often a term measured or documented (US) - College can mean high school or university (NZ) - It can also include diplomas / certs….usually delivered in colleges which is often different from university (Ire) - Associate's degree is also common in the US and is different from trade school - No formal education is one of the common items we use but this could be relative (Bhutan). Indigenous education qualification is another term to capture education in Monks and nuns for example. - Was undergraduate degree considered as wording? - Is the intention to query health literacy or just literacy? - The way the question stem is worded "highest level" is a little problematic implies that the responses are ordered. Not sure that is the intent - maybe can be more about educational background (rather than level) - There are some degrees conferred by universities in Ghana that are not Bachelors. E.g. HND - Yes, in South Africa too - tertiary education or just degree - Probably a term like 'other degree' or 'other tertiary qualification' may be appropriate to capture the diplomas, HNDs and the rest |
| *Proposed item:*  Which of the following best describes your highest level of education?  o No (or limited) formal schooling  o Primary or elementary school  o Secondary or high school  o Vocational or trade school  o Bachelor's degree  o Post-graduate degree (e.g. Master's, Doctorate)  o Other (please specify: ____)  o Prefer not to answer  *Vote result:*  *Strongly agree – 15%*  *Agree – 85%*  *Reservations - 0*  *Disagree - 0* | *Refined Item:*  Variable name: Education  Item: Which of the following best describes your highest level of education?   - No (or limited) formal schooling - Primary or Elementary School - Lower Secondary or Middle School - Upper Secondary or Senior/High School - Technical/Trade/Vocational Training (e.g. Certificate, Diploma, Apprenticeship) - Some Higher Education (College or University) - Undergraduate Degree (e.g. Associate, Bachelor’s Degree) - Postgraduate Degree (e.g. Graduate Certificate or Diploma, Master’s or Doctoral Degree) - Other Education (please specify: ______) - Prefer not to answer |

SOCIOECONOMIC STATUS

| Main discussion points | Summary of ‘chat notes’ |
| --- | --- |
| - Can you expand on what is meant by basic living expenses – keen for healthcare to be included amongst the examples - Food, water quality, medicines, healthcare – all ok. Housing is generic (rent and bills not so globally relevant). Expenses – consider inclusiveness of items. - Able to meet your basic living expenses. Frames it around basic living expenses which may introduce ceiling or floor effects. E.g. someone who is very poor might be able to meet their basic living expenses comfortably but everything else is unavailable to them. What does it mean to meet basic living expenses comfortably? There is a lot of space above basic living expenses. Is the formulation of the question right? Consider removing ‘basic’. - Climate change is a health equity issue – access to clean air and water – gives insights into the ‘place’ category | - Consider having a "it varies" answer to this - Alternative wording: Meet with no difficulty, a little difficulty, some difficulty, much difficulty, cannot meet - For people who may not have financial security this may vary based on when it is asked - Consider not calling this item socioeconomic status as education and work status are also indicators of SEP. Eventually calling it financial/economic status? - "meet adequately" might be difficult to understand and difficult to differentiate from "just meet" and "meet comfortably" - easy to play with the wording though - Agree income will be highly variable even if meet basic living expenses - What about, "How easily can you afford your basic living expenses?" - very easily, somewhat easily, not easily, not at all [something to that effect?] - One of the things we have not covered in basic living is climate change and in countries where access to just the basic necessities e.g. clean water, air pollution issues. - I wonder if we need to list "with help of others" |
| *Proposed item:*  How well are you able to meet your basic living expenses? (e.g. food, rent, medicines, bills)  o Meet comfortably  o Meet adequately  o Just meet  o Cannot meet  o Prefer not to answer  *Vote result:*  *Strongly agree – 15%*  *Agree – 79%*  *Reservations – 3%*  *Disagree – 3%* | *Refined Item:*  Variable name: Financial status  Item: How easily are you able to meet your basic living expenses? (e.g. food, housing, healthcare)   - Can meet very easily - Can meet easily - Can just meet - Can meet sometimes but not always - Cannot meet - Prefer not to answer |

AGE

| Main discussion points | Summary of ‘chat notes’ |
| --- | --- |
| - More common to ask: “What is your age?” (years). How old are you seems to be the phrasing you would use to ask a child. - What is your age doesn’t translate in South Africa (there is no possession of it). How old are you will be more easily translated. - I also preferred the phrasing that asks about age. I wonder if it would be more translatable if it was a statement rather than a question – for example, “Tell us your age in years.” | - In our languages (South African) it is easier to ask how old are you than what is your age. The abstract 'what is your age' is difficult to translate - General agreement with asking “What is your age?” (instead) |
| *Proposed item:*  How old are you? _____ (years)  *Vote result:*  *Strongly agree – 60%*  *Agree – 40%*  *Reservations - 0*  *Disagree - 0* | *Refined Item:*  Variable name: Age  Item: What is your age?   - ____ (years) - Prefer not to answer |

**Appendix 9: Focus group discussion guide**

*Researcher Focus Groups*

Introduction (10 mins):

- Introductions, establish connections
- Provide a brief, general overview of:
  - The purpose of the questionnaire
  - The development process
  - The difference between Item Set A and Item Set B

Provide time to look at the questionnaire before the discussion (5 mins)

Semi-structured questions/discussion guide:

With reference to Set A:

1. What do you think Set A is trying to measure?
2. Do you understand all the questions in Set A?
3. Are the questions (and response categories) clearly worded?
4. Are the response categories relevant to the setting in which you work? Are they appropriate and sufficient (in your context)?
5. Are there any items in Set B that you think some people may feel uncomfortable answering? Or risk being judgemental, intrusive or distressing?
6. Do you have any concerns about their cross-cultural acceptability, relevance, or translatability?
7. What do you think of the length of this questionnaire?

With reference to Set B:

Questions 1 – 6 as for set A

1. How many items from this set do you think it is reasonable to ask?

General questions:

1. Do you have any thoughts, suggestions or concerns related to our aim of achieving widespread adoption of Set A (all items) and Set B (selected items) in all human pain research?
2. Do you have any suggestions for promoting or assisting uptake?
3. Do you have any suggestions related to how we can make this survey simple to administer, access and complete?

*Patient and Public Focus Groups*

Introduction (5-10 mins):

- Introductions
- Brief explanation of the session, including the discussion focus

Participants read/complete the questionnaire (and make notes)

Semi-structured questions/discussion guide:

1. What do you think these questions are trying to assess (in general)?
2. Do you understand all the questions?

Please tell me about any that you weren’t sure about…

1. Are they all worded clearly?

Please tell me about any that weren’t clear…

1. Are the response categories relevant to the setting in which you live?

Are there any that are not relevant or appropriate?

Do you think any additional categories are needed?

1. Did answering any of the questions make you feel uncomfortable in any way?

Or do you think any of them could make anyone else uncomfortable?

1. Do you have any concerns about whether any of these questions might be inappropriate or not relevant in some to some people or cultures?
2. If you were a participant in a research study and you were asked questions like this - how many questions do you think you would be happy to answer?

**Appendix 10: Focus group participant characteristics**

| Characteristic | N (Total n = 40) | Researcher Focus Groups (n = 2) | Patient/Public Focus Groups (n = 5) |
| --- | --- | --- | --- |
| Age |  |  |  |
| 18-30 | 7 | 3 | 4 |
| 31-40 | 8 | 6 | 2 |
| 41-50 | 6 | 3 | 3 |
| 51-60 | 6 | 1 | 5 |
| 61-70 | 3 | - | 3 |
| Missing data | 10 | 7 | 3 |
| Sex |  |  |  |
| Female | 31 | 16 | 15 |
| Male | 9 | 4 | 5 |
| Gender |  |  |  |
| Woman | 25 | 10 | 15 |
| Man | 9 | 4 | 5 |
| Non-binary | 2 | 2 | - |
| Missing data | 4 | 4 | - |
| Country of residence |  |  |  |
| Australia | 7 | 2 | 5 |
| Brazil | 4 | 4 | - |
| Chile | 7 | 2 | 5 |
| India | 4 | - | 4 |
| Nigeria | 2 | - | 2 |
| South Africa | 2 | 1 | 1 |
| United States of America | 3 | 3 | - |
| *1 participant from:* China, France,  Germany, Ireland, Malta, Netherlands,  Peru, Portugal, South Africa, Spain,  Taiwan, United Kingdom, Vietnam | 13 | 8 | 5 |
| Region of residence |  |  |  |
| City | 31 | 14 | 17 |
| Town | 4 | 2 | 2 |
| Rural area | 1 | - | 1 |
| Missing data | 4 | 4 | - |
| Education status |  |  |  |
| Upper secondary | 1 | - | 1 |
| Technical/trade/vocational Training | 3 | - | 3 |
| Some higher education | 2 | - | 2 |
| Undergraduate degree | 5 | 1 | 4 |
| Postgraduate degree | 25 | 15 | 10 |
| Missing data | 4 | 4 | - |
| Financial status (How easily can you meet your basic living expenses?) | | | |
| Can meet very easily | 11 | 10 | 1 |
| Can meet easily | 15 | 5 | 10 |
| Can just meet | 6 | 1 | 5 |
| Can meet sometimes but not always | 3 | - | 3 |
| Cannot meet | 1 | - | 1 |
| Missing data | 4 | 4 | - |

| Working status |  |  |  |
| --- | --- | --- | --- |
| Full time worker | 14 | 12 | 2 |
| Part time worker | 13 | 2 | 11 |
| Not working | 6 | 1 | 5 |
| On disability or medical leave | 5 | 1 | 4 |
| Other (retired, student, self-employed) | 3 | 1 (student) | 2 (retired, self-employed) |
| Missing data | 4 | 4 |  |
| Lived experience of chronic pain |  |  |  |
| Current chronic pain | 23 | 4 | 19 |
| Previous chronic pain (but not current) | 1 | 1 | - |
| Missing data | 5 | 4 | 1 |
| Current chronic health condition |  |  |  |
| Sickle Cell Disease | 3 | 3 | Data not collected |
| Other | 3 | 3 | Data not collected |

**Appendix 11: Focus group summary of results**

| SET A | | Summary of main focus group feedback points (and item modifications) |
| --- | --- | --- |
| General information for researchers | | Further developed in line with focus groups discussions |
| General information for participants | | Modifications made to preamble to expand on reassurance (confidentiality), explanation & rationale |
| A2 | What is your sex? | - Inform researchers about appropriate use of terminology. E.g. researchers who omit the question about gender identity, should use the question "what sex were you assigned at birth" and report this variable as 'sex'. - Changed wording of question (previously: What sex were you assigned at birth) – since it is common for people to discover their intersex status later in life 🡪 also modified the pre-amble to suit. - Changed wording to “A term not listed…” |
| A3 | What term(s) best describe your gender identity? | - Changed wording of question (previously: “How do you describe your gender”) - Changed wording to “An identity not listed…” - Deleted the option: “I don’t know what this question means” (the preamble explains what it means) |
| A4 | Which category best describes where you live? | - Further developed explanations for researchers to: (i) tailor the response options to suit local contexts and (ii) collect data with greater granularity (relevant to research questions) that can be mapped to the 3-tier classification. |
| A5 | Which category best describes how you identify? (in terms of race, ethnicity, or cultural identity) | Major change to the approach to this item based on feedback:   - It seems odd/confusing/inappropriate to mix terms that relate to geography with terms that relate to ethnicity/culture - Lots of items in this list and still imperfect. Unlikely to reach a list that is entirely complete, inclusive, specific, balanced.. - Further developed researcher information in line with focus group discussions - Participants in India and Chile had difficulty identifying a geographical region that applied to them - Self-reported identity was a well-accepted approach - Initially presented examples that were relevant to population/setting however this was frequently confusing 🡪 suggest making this optional (for researchers). 🡪 don’t provide suggestions (just let participants self-describe). - Provide a link to further guidance for developing this item (with examples), and guidance for reporting. |
| A6 | Which of the following best describes your level of education? | - Consider removing ‘’highest’’ (level of education) from question: It doesn't really add anything to the question and if some people didn't complete the education level it might be confusing. - This item generated much discussion. In Chile – the system is structured/labelled quite differently 🡪 added in the brackets indicating the number of years of schooling that assisted translatability. |
| A7 | How easily are you able to meet your basic living expenses? (e.g. food, housing, healthcare) | - Some discussion that it may not accurately define SES, e.g. someone in a well-off area may say that they can only just meet… or spend lots of money on alcohol or cigarettes and not have much money left to spend on living expenses... - Consider adding a free-text comment box for participants to provide explanation |
| A8 | What best describes your current work situation? | - Added “paid” to question; added: “A work situation not listed…” - Changed sub-question (previously: “Select any reasons for not working you would like to provide”) - Consider also adding examples e.g. casual work, informal work, unregulated work. - Changed ‘On disability leave’ to “Disability or health reasons” (‘leave’ implies someone has a job to go back to) - Consider adding “I am looking for work” to the list of reasons |

| SET B | | Summary of main focus group feedback points (and item modifications) |
| --- | --- | --- |
| General Information for researchers: | | - Developed information/explanation - Prompted researchers to consider their rationale for collecting the data (and what they will do with it) |
| DISCRIMINATION | |  |
| B1 | Have you been treated or judged unfairly *in society** due to any of the following reasons? | - Participant suggestion to add: “in society” which I agree offers further question clarification - Suggest option to replace “in society” with “in a healthcare setting” and including one or both items - Re-positioned this item at the top of Set B (removed it from the plus category at the end) - Added ‘or other chronic health condition’ to the Disability category - Added the 2^nd^ part to the question to quantify degree of discrimination |
| PLACE | |  |
| B2 | How easy is it for you to access the health care services you need? | - Some discussions around the ambiguity of this question. E.g. what types of services? What sort of access difficulties is it referring to? (e.g. transport related, waiting times, types of services…). - Consider if there is benefit to this open question. |
| B3 | Do you have access safe and reliable basic amenities where you live? | - Deleted “facilities” from “toilet facilities” (in example) – easier to translate and may be easier to understand. - Added “where you live” to the question - Added “yes,” and “no,” to response options and left “Rarely” as is. |
| B4 | Do you feel safe in the area where you live? | - Previously: Do you feel safe in your neighbourhood? But ‘neighbourhood’ isn’t a term that is universally used. (OR, could keep neighbourhood and researchers can adapt it if it doesn’t fit… - People may not understand why they are being asked this question 🡪 Added to the Info for Participants: “You may be unsure why some of the questions are important or feel that they are not relevant to you…” |
| B5 | How satisfied are you with your housing conditions? | - Previous question: “How satisfied are you with the conditions in which you live? (without examples) was considered a bit vague. Re-worded question and added some examples to be clearer what it is referring to. - Is the middle response the best option? |
| B6 | Which best describes your current living situation? | - Inserted “usually” into option 4; inserted “regularly” into option 5; added the final option - Deleted “e.g. homeless” from option 5 (it is an ambiguous term and could apply to both option 4 & 5) |
| B7 | Do you have access to transport that can reliably get you to where you need to go? | - Changed question: “transportation” to “transport” - Safety is an issue in some cultures. Could say: “reliably and safely” (but leave as is for the sake of providing a simpler question that is relevant in most contexts) |
| B8 | How easy is it for you to get to open space? | - Previously: How easy is it for you to get to open space with trees or other vegetation? 🡪 The second half isn’t required if examples are provided. |
| RACE, ETHNICITY, CULTURE & LANGUAGE | |  |
| B9 | Are you able to communicate easily in a main language spoken where you live? | - Previous question: “Are you able to communicate easily (speak and/or write) in the main language spoken where you live?” – deleted (speak and/or write) - it is a bit limiting (also “understand”?) and not required. - Changed “the” to “a” – acknowledging that some countries have more than one main language. |
| OCCUPATION | |  |
| B11 | How satisfied are you with your current (paid) work situation? | - Consider replacing “not satisfied or unsatisfied” with “neutral" |
| B12 | Does your (paid) work include access to health benefits including insurance, or compensation? | - Added option 4 - A South African wouldn’t understand ‘insurance’ – it is called medical aid. Left unchanged, this is a context-specific adaptation that can be made. |
| B13 | Does your (paid) work involve moderate or high health risks? | - Deleted ‘or well-being’ from the question examples – not readily understood in all contexts. - Added some differentiation between types of risks (physical or mental health or both) |
| B14 | What best describes your current main occupation/paid job?* | - Response options mostly ok, but sometimes difficult to find an option that fits. Added: “A job not listed…” |
|  | | |
| B15 | Have your religious beliefs and/or practices impacted your health care? | - Previous question: “negatively impacted” health care. Deleted “negative”. - Extended the response categories to determine if positive or negative impact |
| B16 | What is your religion? | - Changed “other” to “A religion not listed…” |
| EDUCATION | |  |
| B18 | Which of the following best describes the level of education of your parent(s)? | - Previously: …. “of your mother or father? (whichever is the highest)” 🡪 this assumes participants were raised by a mother and father. |
| SOCIOECONOMIC POSITION | |  |
| B19 | In the past 6 months, how much has financial cost stopped you from accessing healthcare or medications? | - Added “financial” (there are all sorts of ‘costs’) |
| B20 | Do you receive financial/basic needs support from government or community programs? | - Changed response options from ‘Always’ to ‘never’, to refer to regularity. Seemed a better way of capturing how peoples access to supports. |
| SOCIAL CAPITAL | |  |
| B24 | Which of the following best describes your living arrangements? | - Changed ‘sole parent’ to ‘single parent’ - Changed ‘other’ to ‘A living arrangement not listed..’ |
| PLUS | |  |
| B28 | Do you (and your family) have reliable access to the internet when you need it? | - Added “whenever you need it” which indicates this question refers not just to workplace.. |
| B30 | What is your current sexual orientation? | - Further consultation with Codie Primeau a Canadian Researcher and member of the Queer community - 🡪 Separated gay and lesbian; Suggested a broader range of response options, including: Asexual, Demisexual, Heteroflexible, Homoflexible, Pansexual, Queer. - Acknowledged potential for changes in appropriate language and terms over time. |

**Appendix 12: ISSHOOs Set A – Arabic***

*Note: This translation has been machine-generated using Google Translate. A validated translation is being developed and will be available on the ISSHOOs website when completed ([www.isshoos.org](http://www.isshoos.org))

*ملاحظة: تم توليد هذه الترجمة آليًا باستخدام جوجل ترانسليت. يجري حاليًا تطوير ترجمة معتمدة، وستكون متاحة على موقع ISSHOO الإلكتروني عند اكتمالها ([www.isshoos.org](http://www.isshoos.org)

| مجموعة ISSHOOs A | | | |
| --- | --- | --- | --- |
| 8 عناصر يوصى بإدراجها في جميع أبحاث الألم البشري التي تشمل مشاركين بالغين | | | |
| تهدف المجموعة (أ) إلى توجيه عملية جمع *البيانات الاجتماعية والديموغرافية بشكل موحد،* مما يُمكّن من وصف المشاركين في أبحاث الألم من خلال مجموعة من الخصائص المعروفة بتأثيرها على الصحة والإنصاف الصحي. بالإضافة إلى المجموعة (أ)، يُرجى مراعاة ما يلي:   - العناصر الاجتماعية والديموغرافية الإضافية ذات الصلة ببيئة الدراسة والسكان و/أو أسئلة البحث. - مجموعة فرعية من العناصر من مجموعة ISSHOOs B.   لجميع عناصر المجموعة أ :   1. قم بتخصيص اللغة أو فئات الاستجابة لتناسب مجتمع الدراسة/البيئة (حسب الحاجة). 2. احذف أي فئات استجابة غير ذات صلة أو مناسبة لمجتمع الدراسة/الإعداد. | | | |
| معلومات للمشاركين (موصى بها):  *تتطلب الأسئلة التالية بعض المعلومات الشخصية (وهذا أمر شائع في الأبحاث الصحية). ستُحفظ إجاباتك في مكان آمن، وسيتم الإبلاغ عنها كـ "إجابات جماعية"، مما يعني أنه لن يتمكن أحد من ربط إجاباتك بك.*  *قد لا تكون متأكدًا من أهمية بعض الأسئلة أو تعتقد أنها غير ذات صلة بك. من المرجح أن تُستخدم المعلومات التي تقدمها لإعداد ملخص للأشخاص المشاركين في هذا البحث. كما قد يُسهم ذلك في زيادة فهم كيفية تأثير العوامل الشخصية وخلفيات الأشخاص وحياتهم اليومية على صحتهم.* | | | |
| أ1 . العمر | | | |
| ما هو عمرك؟ | - ____ (سنين) - أفضل عدم الإجابة | | |
| أ2. الجنس | | | |
| ملحوظة :   1. في بعض إعدادات الدراسة قد يكون من المناسب تغيير أو استبعاد مقدمة الجنس (أدناه) وتعديل فئات الاستجابة لأسباب سياسية أو ثقافية أو دينية أو تتعلق بالسلامة أو لأسباب أخرى. | | | |
| مقدمة عن "الجنس" للمشاركين (موصى بها):  *الجنس هو تسمية (عادةً ما يكون ذكرًا أو أنثى) تُمنح للطفل عند ولادته، بناءً على أجزاء جسمه. قد تكون هناك اختلافات في الجنس، مثل "الخنثى" الذي قد يُحدد عند الولادة أو لاحقًا.* | | | |
| ما هو جنسك؟ | - أنثى - ذكر - خنثى - مصطلح غير مدرج (يرجى التحديد: ____) - أفضل عدم الإجابة | | |
| أ3. الهوية الجنسية | | | |
| ملحوظة :   1. في بعض إعدادات الدراسة قد يكون من المناسب تغيير المقدمة أو استبعادها وتعديل فئات الاستجابة لأسباب سياسية أو ثقافية أو دينية أو أمنية أو أسباب أخرى. 2. عندما يختار الباحثون حذف السؤال حول الهوية الجنسية، فإننا نوصي بإدراج العنصر A2 (أعلاه) والإبلاغ عن المتغير "الجنس". 3. خذ بعين الاعتبار توفير مجموعة أوسع من فئات الاستجابة التي تسترشد بالمجتمع المتنوع بين الجنسين (ذات الصلة بمشاركين دراستك، وأسئلة البحث وكما هو مناسب في سياق دراستك). | | | |
| مقدمة حول "الهوية الجنسية" للمشاركين (موصى بها):  *الهوية الجنسية هي الإحساس الشخصي بجنس الشخص - سواءً كان رجلاً، امرأة، كليهما، لا هذا ولا ذاك، أو أي شيء آخر. وهي شخصية، وقابلة للتغير مع مرور الوقت، وقد تكون أو لا تكون مطابقة لجنس الشخص.* | | | |
| ما هي المصطلحات التي تصف هويتك الجنسية بشكل أفضل؟ *حدد كل ما ينطبق.* | - امرأة - رجل - غير ثنائي - مصطلح غير مدرج (يرجى التحديد: ____) - أنا لست متأكدًا، أو أنا أتساءل - أفضل عدم الإجابة | | |
| أ4. مكان الإقامة | | | |
| ملحوظة :   1. عند الإمكان، اجمع بيانات متوافقة مع التصنيف العالمي التالي لدرجة التحضر. صمّم فئات الاستجابة بما يتناسب مع بيئة دراستك، واستخدم مصطلحات مناسبة لمجتمع دراستك. يتوفر المزيد من الإرشادات لإعداد هذا العنصر وإعداد التقارير عنه في مخطوطة منفصلة ^.^  \|  \| مصطلحات قصيرة \| المصطلحات التقنية \| \| --- \| --- \| --- \| \| المستوى 1: \| مدينة \| منطقة ذات كثافة سكانية عالية \| \|  \| مدينة \| مستوطنة كبيرة \| \| المستوى الثاني: \| المدينة والمنطقة شبه الكثيفة \| منطقة الكثافة المتوسطة \| \|  \| مدينة كثيفة \| مستوطنة كثيفة ومتوسطة \| \|  \| مدينة شبه كثيفة \| مستوطنة متوسطة الكثافة وشبه كثيفة \| \|  \| منطقة ضواحي أو شبه حضرية \| منطقة شبه كثيفة \| \| المستوى 3: \| منطقة ريفية \| منطقة ذات كثافة سكانية منخفضة \| \|  \| قرية \| مستوطنة صغيرة \| \|  \| منطقة ريفية متفرقة \| منطقة ذات كثافة منخفضة \| \|  \| منطقة غير مأهولة بالسكان في الغالب \| منطقة ذات كثافة سكانية منخفضة جدًا \|  1. فكّر في إضافة فئات استجابة إضافية لجمع بيانات أكثر تفصيلًا، مرتبطة بمجتمع دراستك، وبيئة البحث، وأهدافه. اربطها بالتصنيف ثلاثي المستويات (انظر المثال أدناه). 2. يمكن للباحثين، بدلاً من ذلك أو بالإضافة إلى ذلك، جمع الرمز البريدي للمشاركين في البحث واستخدام هذه البيانات لتصنيف مكان الإقامة. | | | |
| ما هي الفئة التي تصف المكان الذي تعيش فيه بشكل أفضل؟ | | - مدينة - بلدة - منطقة ريفية - أفضل عدم الإجابة | - مستوطنة أو بلدة غير رسمية *(المستوى 1)* - مدينة *(المستوى 1)*   **Example:** (South Africa)   - المدينة *(المستوى 2)* - قرية *(المستوى 3)* - منطقة ريفية *(المستوى 3)* |
| أ5. العرق والانتماء العرقي والهوية الثقافية | | | |
| ملحوظة :   1. سيُمكّن هذا العنصر من تحديد العرق والانتماء العرقي والهوية الثقافية للمشاركين في الدراسة. نوصي بإنشاء فئات استجابة تُمكّن من تحديد الفئات الفرعية من مجتمع الدراسة، وخاصةً أولئك الذين قد يعانون من التهميش أو الحرمان. 2. فكر في إزالة هذا العنصر إذا كنت تعتقد أنه سيسبب إساءة خطيرة، أو إذا كان يتعارض مع القوانين أو اللوائح أو العادات المحلية أو الإقليمية أو الوطنية. 3. من المهم أن نكون حساسين تجاه الفروق الثقافية:  - قم بتخصيص العنصر (العناصر) وفقًا للبيئة التي تُجرى فيها الدراسة مع مراعاة تنوع خلفيات مجموعة الدراسة بأكملها (وليس فقط المجموعات الأكثر شيوعًا). - عندما يكون ذلك ممكنا، استخدم المصطلحات والفئات التي تم استخلاصها في البلد الذي تُجرى فيه الدراسة والتي يعتبرها السكان المحليون مقبولة. - مصطلحات "العرق" و"الانتماء العرقي" و"الهوية الثقافية" ليست مترادفة. قد تختلف معانيها وقبولها باختلاف السياقات. استخدم المصطلحات الأنسب لبيئة دراستك.   البند أ5 (أ): سؤالان موصى بهما للأبحاث التي تشمل مشاركين من بلدان متعددة.  البند أ5 (ب): النهج الموصى به للبحث في بلد واحد أو عدد قليل من البلدان المختارة. | | | |
| أ5(أ). العرق، والانتماء العرقي، و/أو الهوية الثقافية ( دراسة *متعددة البلدان)* | | | |
| أوصى بسؤالين: (أ) *و* (ب) | | | |
| السؤال (أ):  في أي بلد تعيش؟ | | - توفير مربع استجابة نصية مجانية   *أو*  قائمة منسدلة لجميع البلدان (استطلاع عبر الإنترنت)   - أفضل عدم الإجابة | |
| *و* السؤال (ii):  صف كيفية تحديد هويتك من حيث العرق أو العرق أو الهوية الثقافية. | | *قم بتوفير ما يصل إلى 3 مصطلحات تصف بشكل أفضل كيفية تحديدك لما يلي:*   - _________________________________ - أفضل عدم الإجابة | |
| أ٥(ب). العرق، والانتماء العرقي، و/أو الهوية الثقافية ( دراسة دولة *واحدة )* | | | |
| ملحوظة :   1. تم توضيح النهج المفضل لجمع البيانات أدناه. 2. يمكنكم الاطلاع على إرشادات وأمثلة خاصة بكل بلد على [الموقع www.isshoos.org](http://www.isshoos.org) . يُنصح باستخدام الأمثلة المُقدمة لتسهيل توحيد جمع البيانات وإعداد التقارير. 3. قد يكون من المناسب تقديم خيار الاستجابة للنص الحر - راجع السؤال (ii) (أعلاه). | | | |
| ما هي الفئة التي تصف بشكل أفضل كيفية تعريفك؟ | *تطوير فئات الاستجابة ذات الصلة ببيئة الدراسة المحددة.*   - *جمع المعلومات حول العرق والإثنية و/أو الهوية الثقافية بأكبر قدر ممكن من التفاصيل.* - *استخدم مصطلحات مناسبة ومقبولة لمجتمع الدراسة. فكّر في تطبيق أو تعديل الفئات المدرجة في مسوحات التعداد الوطني (إن وجدت).* - *يتم توفير إرشادات إضافية لإنشاء هذا العنصر والإبلاغ عنه في مخطوطة منفصلة "الشرح والتوضيح" ^وعلى^ موقع ISSHOOs الإلكتروني (* [*www.isshoos.org*](http://www.isshoos.org) *).* | | |
| أ6. التعليم | | | |
| أي مما يلي يصف بشكل أفضل أعلى مستوى تعليمي لديك؟ | - قلة أو عدم وجود تعليم رسمي (3 سنوات من التعليم أو أقل) - المدرسة الابتدائية أو الإعدادية (4 - 8 سنوات من التعليم) - المرحلة الثانوية أو المتوسطة (8-10 سنوات من التعليم) - المرحلة الثانوية العليا أو الثانوية (11 - 13 سنة من التعليم) - التدريب الفني/التجاري/المهني (على سبيل المثال الشهادة، الدبلوم، التدريب المهني) - بعض التعليم العالي (كلية أو جامعة) - درجة البكالوريوس (على سبيل المثال درجة البكالوريوس) - درجة الدراسات العليا (مثل الماجستير والدكتوراه) - مستوى التعليم غير المذكور (يرجى التحديد: ______) - أفضل عدم الإجابة | | |
| أ7. الوضع المالي | | | |
| ما مدى سهولة قدرتك على تحمل نفقات معيشتك الأساسية؟ (مثل الطعام، السكن، الرعاية الصحية) | - يمكن تحمله بسهولة شديدة - يمكن تحمله بسهولة - لا أستطيع تحمل التكلفة إلا بالكاد - لا أستطيع تحمل التكاليف - أفضل عدم الإجابة | | |
| أ8. حالة العمل | | | |
| ما هو أفضل وصف لوضع عملك الحالي المدفوع الأجر؟  ** ملحوظة :*  *تضمين سؤال إضافي* | - عامل بدوام كامل - عامل بدوام جزئي * - لا يعمل * - موقف عمل غير مدرج (يرجى التحديد: ___ _) * - أفضل عدم الإجابة   *اختر أي أسباب لموقف عملك الذي ترغب في تقديمه:  *حدد كل ما ينطبق.*   - - أسباب الإعاقة أو الصحة   - الوالد/مدير المنزل/مقدم الرعاية "في المنزل"   - طالب   - متقاعد   - غير قادر على العثور على عمل   - سبب غير مدرج (يرجى التحديد: ____)   - غير ذي صلة/أفضل عدم الإجابة | | |

مخطوطة "الشرح والتوضيح" ^لـ^ ISSHOOs في طور التنفيذ

**Appendix 13: ISSHOOs Set A – Chinese***

*Note: This translation has been machine-generated using Google Translate. A validated translation is being developed and will be available on the ISSHOOs website when completed ([www.isshoos.org](http://www.isshoos.org))

*注：此翻译由 Google 翻译机器生成。经过验证的翻译正在开发中，完成后将在 ISSHOOs 网站上发布（[www.isshoos.org](http://www.isshoos.org)

| ISSHOOs 套装 A | | | |
| --- | --- | --- | --- |
| 建议在所有涉及成人参与者的人类疼痛研究中纳入 8 个项目 | | | |
| 数据集 A 的目的是指导以标准化方式收集*最低限度的社会人口统计数据，*以便能够描述疼痛研究参与者的一系列已知会影响健康和健康公平的特征。除了数据集 A 之外，还请考虑包括：   - 与您的研究环境、人口和/或研究问题相关的其他社会人口统计项目。 - ISSHOOs B 组项目子集。   对于所有 A 组商品：   1. 定制语言或响应类别以适合研究人群/环境（根据需要）。 2. 删除任何与研究人群/环境不相关或不适当的响应类别。 | | | |
| 参会人员须知（推荐）：  *以下问题会询问一些个人信息（这在健康研究中很常见）。您的答案将被保密，并将被报告为“集体答案”——这意味着没有人可以将您的答案与您联系起来。*  *您可能不确定为什么某些问题很重要，或者认为这些问题与您无关。您提供的信息可能会用于报告参与这项研究的人员的摘要。它还可能有助于增进对个人因素、人们的背景及其日常生活如何影响健康的理解。* | | | |
| A1 . 年龄 | | | |
| 你几岁？ | - ____ （年） - 不想回答 | | |
| A2.性别 | | | |
| 笔记：   1. 在某些研究环境中，由于政治、文化、宗教、安全或其他原因，改变或排除性别序言（如下）并修改回答类别可能是最合适的。 | | | |
| 参与者的“性”序言（推荐）：  *性别是婴儿出生时根据其身体部位赋予的标签（最常见的是男性或女性）。性别可能存在差异，例如“双性人”可能是在出生时或以后被赋予的。* | | | |
| 您的性别是？ | - 女性 - 男性 - 双性人 - 未列出的术语（请说明：____） - 不想回答 | | |
| A3.性别认同 | | | |
| 笔记：   1. 在某些研究环境中，由于政治、文化、宗教、安全或其他原因，改变或删除序言并修改回答类别可能是最合适的。 2. 如果研究人员选择省略有关性别认同的问题，我们建议包括项目 A2（上文）并报告变量“性别”。 3. 考虑提供由性别多元化社区提供的更广泛的响应类别（与您的研究参与者、研究问题相关并适合您的研究环境）。 | | | |
| 参与者的“性别认同”序言（推荐）：  *性别认同是个人对自己性别的感受，无论是男性、女性、男性和女性、女性和男性之外的其他性别。性别认同是个人的，会随着时间而改变，可能与个人的性别相同，也可能不同。* | | | |
| 哪些术语最能描述您的性别认同？ *选择所有适用项。* | - 女士 - 男人 - 非二元性别 - 未列出的术语（请说明：____） - 我不确定，或者我在质疑 - 不想回答 | | |
| A4.居住地 | | | |
| 笔记：   1. 尽可能收集符合以下全球城市化程度分类的数据。根据您的研究环境定制响应类别，并使用与您的研究人群相关的术语。有关生成和报告此项目的进一步指导在单独的手稿^a^中提供。  \|  \| 短期 \| 技术术语 \| \| --- \| --- \| --- \| \| 第 1 层： \| 城市 \| 人口稠密地区 \| \|  \| 城市 \| 大型定居点 \| \| 第 2 级： \| 城镇和半密集地区 \| 中等密度区 \| \|  \| 密集的城镇 \| 密集、中等沉降 \| \|  \| 半密集城镇 \| 半密实、中等沉降 \| \|  \| 郊区或近郊地区 \| 半密集区 \| \| 第 3 级： \| 农村 \| 人口稀少地区 \| \|  \| 村庄 \| 小型定居点 \| \|  \| 分散的农村地区 \| 低密度区 \| \|  \| 大部分无人居住的地区 \| 极低密度地区 \|  1. 考虑包括额外的响应类别，以收集与您的研究人群、设置和研究目标相关的更详细的数据。将它们“映射”到 3 层分类（参见下面的示例）。 2. 研究人员还可以另外或另外收集研究参与者的邮政编码，并使用这些数据对居住地进行分类。 | | | |
| 哪个类别最能描述您的居住地？ | | - 城市 - 镇 - 农村 - 不想回答 | - 非正式定居点或乡镇 *（第 1 级）* - 城市 *（第 1 级）*   **Example:** (South Africa)   - 城镇*（2 级）* - 村庄 *（第 3 级）* - 农村 *（第 3 级）* |
| A5.种族、民族和/或文化认同 | | | |
| 笔记：   1. 此项将使报告研究参与者的种族、民族和/或文化身份成为可能。我们建议生成响应类别，以便识别研究人群的子群体 - 特别是那些可能遭受边缘化和/或不利影响的人。 2. 如果您认为此项目会引起严重冒犯，或者它违反当地、地区或国家法律、法规或习俗，请考虑删除此项目。 3. 对文化差异保持敏感很重要：  - 根据研究环境定制项目，并考虑整个研究人群（而不仅仅是最常见的群体）的背景多样性。 - 在可能的情况下，请使用研究所在国家/地区产生的、当地人可以接受的术语和类别。 - “种族”、“民族”和“文化认同”这几个术语不可互换。它们在不同的环境中可能具有不同的含义和不同的可接受性。请使用最适合您研究环境的术语。   项目 A5(a)：针对涉及多国参与者的研究建议提出的两个问题。  项目 A5(b)：针对单个国家或少数选定国家进行研究的推荐方法。 | | | |
| A5(a).种族、民族和/或文化认同（*多*国研究） | | | |
| 推荐 2 个问题：(i)*和*(ii) | | | |
| 问题（一）：  您居住在哪个国家？ | | - 提供自由文本响应框   *或者*  所有国家/地区的下拉列表（在线调查）   - 不想回答 | |
| *和*问题 (ii)：  描述您如何从种族、民族或文化身份方面进行识别。 | | *请提供最多 3 个术语来最准确地描述您的身份：*   - _________________________________ - 不想回答 | |
| A5(b).种族、民族和/或文化认同（*单一*国家研究） | | | |
| 笔记：   1. 收集数据的首选方法概述如下。 2. [www.isshoos.org](http://www.isshoos.org)上找到。建议使用所提供的示例来促进数据收集和报告的标准化。 3. 提供自由文本响应选项可能被视为最合适的 - 参见问题（ii）（上文）。 | | | |
| 哪个类别最能描述您的身份？ | *制定与特定研究环境相关的响应类别。*   - *尽可能详细地收集有关种族、民族和/或文化身份的信息。* - *使用与研究人群相关且可接受的术语。考虑应用或调整国家人口普查调查中包含的类别（如果有）。* - *单独的“解释和阐述”手稿^a^和 ISSHOOs 网站（* [*www.isshoos.org*](http://www.isshoos.org) *）中提供了有关此项目的生成和报告的进一步指导。* | | |
| A6.教育 | | | |
| 以下哪项最能描述您的最高教育水平？ | - 很少或没有接受过正规学校教育（3 年或以下） - 小学（4 - 8 年教育） - 初中或中学（8 - 10 年教育） - 高中或高中（11 - 13 年教育） - 技术/贸易/职业培训（例如证书、文凭、学徒制） - 一些高等教育（学院或大学） - 本科学位（例如学士学位） - 研究生学位（如硕士、博士） - 未列出的教育程度（请注明：______） - 不想回答 | | |
| A7.财务状况 | | | |
| 您能轻松负担（支付）基本生活开支吗？ （例如食品、住房、医疗保健） | - 很容易负担得起 - 可以轻松负担 - 仅能负担得起 - 负担不起 - 不想回答 | | |
| A8.工作状态 | | | |
| 以下哪项最能描述您目前的有偿工作状况？  **笔记：*  *包含附加问题* | - 全职工作者 - 兼职工作者* - 不工作* - 未列出的工作情况（请说明：___ _） * - 不想回答   *选择您想要提供的工作情况的原因：  *选择所有适用项。*   - - 残疾或健康原因   - “居家”父母/家庭管理者/看护者   - 学生   - 已退休   - 找不到工作   - 未列出的原因（请说明：____）   - 不相关/不想回答 | | |

ISSHOOs的“解释和阐述”^手稿^ 正在进行中

**Appendix 14: ISSHOOs Set A – French***

*Note: This translation has been machine-generated using Google Translate. A validated translation is being developed and will be available on the ISSHOOs website when completed ([www.isshoos.org](http://www.isshoos.org))

*Remarque: Cette traduction a été générée automatiquement avec Google Traduction. Une traduction validée est en cours de développement et sera disponible sur le site web d'ISSHOOs une fois finalisée ([www.isshoos.org](http://www.isshoos.org)

| ISSHOOs Ensemble A | |
| --- | --- |
| 8 éléments recommandés pour inclusion dans toutes les recherches sur la douleur humaine impliquant des participants adultes | |
| L'objectif de l'ensemble A est de guider la collecte standardisée d'un *ensemble minimal de données sociodémographiques,* permettant de décrire les participants à la recherche sur la douleur selon un éventail de caractéristiques connues pour avoir un impact sur la santé et l'équité en santé. En complément de l'ensemble A, envisagez d'inclure :   - Éléments sociodémographiques supplémentaires pertinents pour votre contexte d’étude, votre population et/ou vos questions de recherche. - Un sous-ensemble d'éléments de l'ensemble B d'ISSHOO.   Pour tous les articles de l'ensemble A :   1. Adaptez la langue ou les catégories de réponses en fonction de la population/du contexte étudié (selon les besoins). 2. Supprimez toutes les catégories de réponses qui ne sont pas pertinentes ou appropriées pour la population/le contexte étudié. | |
| Informations pour les participants (recommandé) :  *Les questions suivantes demandent des informations personnelles (ce qui est courant dans la recherche en santé). Vos réponses seront conservées en toute sécurité et seront enregistrées comme « réponses de groupe », ce qui signifie que personne ne pourra les associer à vous.*  *Vous pourriez ne pas comprendre l'importance de certaines questions ou penser qu'elles ne vous concernent pas. Les informations que vous fournirez serviront probablement à présenter un résumé des personnes impliquées dans cette recherche. Elles pourront également contribuer à mieux comprendre l'influence des facteurs personnels, des antécédents et du quotidien des personnes sur la santé.* | |
| A1 . ÂGE | |
| Quel âge avez-vous? | - ____ (années) - Je préfère ne pas répondre |
| A2. SEXE | |
| Note :   1. Dans certains contextes d’étude, il peut être plus approprié de modifier ou d’exclure le préambule relatif au sexe (ci-dessous) et de modifier les catégories de réponses pour des raisons politiques, culturelles, religieuses, de sécurité ou autres. | |
| Préambule « Sexe » pour les participants (recommandé) :  *Le sexe est une étiquette (généralement masculine ou féminine) attribuée à un bébé à la naissance, en fonction des parties de son corps. Il peut exister des variations de sexe, par exemple « intersexué », qui peuvent être attribuées à la naissance ou plus tard dans la vie.* | |
| Quel est ton sexe ? | - Femelle - Mâle - Intersex - Un terme non répertorié (veuillez préciser : ____) - Je préfère ne pas répondre |

| A3. IDENTITÉ DE GENRE | | | |
| --- | --- | --- | --- |
| Note :   1. Dans certains contextes d’étude, il peut être plus approprié de modifier ou d’exclure le préambule et de modifier les catégories de réponses pour des raisons politiques, culturelles, religieuses, de sécurité ou autres. 2. Lorsque les chercheurs choisissent d’omettre la question sur l’identité de genre, nous recommandons d’inclure l’élément A2 (ci-dessus) et de signaler la variable « Sexe ». 3. Envisagez de fournir une gamme plus large de catégories de réponses informées par la communauté diversifiée en matière de genre (pertinentes pour les participants à votre étude, les questions de recherche et selon le contexte de votre étude). | | | |
| Préambule « Identité de genre » pour les participants (recommandé) :  *L'identité de genre est la perception personnelle de son propre genre – qu'il s'agisse d'un homme, d'une femme, des deux, d'aucun des deux, ou d'un autre. Elle est personnelle, peut évoluer avec le temps et peut être ou non identique au sexe d'une personne.* | | | |
| Quel(s) terme(s) décrit(ent) le mieux votre identité de genre ? *Sélectionnez tout ce qui s'applique.* | - Femme - Homme - Non binaire - Un terme non répertorié (veuillez préciser : ____) - Je ne suis pas sûr, ou je me pose des questions - Je préfère ne pas répondre | | |
| A4. LIEU DE RÉSIDENCE | | | |
| Note :   1. Dans la mesure du possible, collectez des données conformes à la classification mondiale suivante du degré d'urbanisation. Adaptez les catégories de réponses au contexte de votre étude et utilisez des termes pertinents pour la population étudiée. Des instructions complémentaires pour la génération et la communication de cet élément sont fournies dans un manuscrit séparé ^.^  \|  \| Courts termes \| Termes techniques \| \| --- \| --- \| --- \| \| Niveau 1 : \| Ville \| Zone densément peuplée \| \|  \| Ville \| Grande colonie \| \| Niveau 2 : \| Ville et zone semi-dense \| Zone de densité intermédiaire \| \|  \| Ville dense \| peuplement dense et moyen \| \|  \| Ville semi-dense \| Semi-dense, tassement moyen \| \|  \| Zone suburbaine ou périurbaine \| Zone semi-dense \| \| Niveau 3 : \| Zone rurale \| Zone peu peuplée \| \|  \| Village \| Petite colonie \| \|  \| Zone rurale dispersée \| Zone de faible densité \| \|  \| Zone en grande partie inhabitée \| Zone à très faible densité \|  1. Envisagez d'inclure des catégories de réponses supplémentaires pour recueillir des données plus détaillées, adaptées à la population étudiée, au contexte et aux objectifs de recherche. Associez-les à la classification à trois niveaux (voir l'exemple ci-dessous). 2. Les chercheurs peuvent également, ou en plus, collecter le code postal des participants à la recherche et utiliser ces données pour classer le lieu de résidence. | | | |
| Quelle catégorie décrit le mieux l’endroit où vous vivez ? | | - Ville - Ville - Zone rurale - Je préfère ne pas répondre | - Établissement informel ou township *(Niveau 1)* - Ville *(Niveau 1)*   **Example:** (South Africa)   - Ville *(Niveau 2)* - Village *(Niveau 3)* - Zone rurale *(Niveau 3)* |
| A5. RACE, ETHNIE et/ou IDENTITÉ CULTURELLE | | | |
| Note :   1. Cet élément permettra de déclarer l'origine ethnique et/ou l'identité culturelle des participants à l'étude. Nous recommandons de générer des catégories de réponses permettant d'identifier les sous-groupes de la population étudiée, en particulier ceux susceptibles d'être marginalisés et/ou défavorisés. 2. Envisagez de retirer cet article si vous pensez qu'il pourrait constituer une offense grave ou s'il contredit les lois, réglementations ou coutumes locales, régionales ou nationales. 3. Il est important d’être sensible aux nuances culturelles :  - Adaptez les éléments au contexte dans lequel l’étude est menée et tenez compte de la diversité des origines de l’ensemble de la population étudiée (et pas seulement des groupes les plus courants). - Dans la mesure du possible, utilisez des termes et des catégories qui ont été élaborés dans le pays dans lequel l’étude est menée et qui sont considérés comme acceptables par la population locale. - Les termes « race », « ethnicité » et « identité culturelle » ne sont pas interchangeables. Leur signification et leur acceptabilité peuvent varier selon le contexte. Utilisez les termes les plus appropriés à votre contexte d'étude.   Point A5(a) : Deux questions recommandées pour les recherches impliquant des participants de plusieurs pays.  Point A5(b) : Approche recommandée pour la recherche dans un seul pays ou dans un petit nombre de pays sélectionnés. | | | |
| A5(a). RACE, ETHNIE et/ou IDENTITÉ CULTURELLE ( Étude *multi* -pays) | | | |
| Recommander 2 questions : (i) *ET* (ii) | | | |
| Question (i) :  Dans quel pays vis-tu ? | | - Fournir une zone de réponse en texte libre   *OU*  Une liste déroulante de tous les pays (enquête en ligne)   - Je préfère ne pas répondre | |
| *ET* Question (ii) :  Décrivez comment vous vous identifiez en termes de race, d’origine ethnique ou d’identité culturelle. | | *Fournissez jusqu'à 3 termes qui décrivent le mieux comment vous vous identifiez :*   - _________________________________ - Je préfère ne pas répondre | |
| A5(b). RACE, ETHNIE et/ou IDENTITÉ CULTURELLE ( Étude *sur un seul* pays) | | | |
| Note :   1. L’approche privilégiée pour la collecte de données est décrite ci-dessous. 2. Des conseils et des exemples spécifiques à chaque pays sont disponibles sur [www.isshoos.org](http://www.isshoos.org) . Il est recommandé d'utiliser les exemples fournis afin de faciliter la normalisation de la collecte et de la communication des données. 3. Il peut être considéré comme plus approprié de proposer une option de réponse en texte libre - voir la question (ii) (ci-dessus). | | | |
| Quelle catégorie décrit le mieux la façon dont vous vous identifiez ? | *Développer des catégories de réponses adaptées au contexte d’étude spécifique.*   - *Recueillez des informations sur la race, l’origine ethnique et/ou l’identité culturelle de la manière la plus détaillée possible.* - *Utiliser des termes pertinents et acceptables pour la population étudiée. Envisager d'appliquer ou d'adapter les catégories figurant dans les recensements nationaux (si disponibles).* - *Des conseils supplémentaires pour la génération et le reporting de cet élément sont fournis dans un manuscrit séparé « Explication et élaboration » ^et^ sur le site Web de l'ISSHOO (* [*www.isshoos.org*](http://www.isshoos.org) *).* | | |
| A6. ÉDUCATION | | | |
| Laquelle des propositions suivantes décrit le mieux votre niveau d’éducation le plus élevé ? | - Peu ou pas de scolarité formelle (3 ans d’études ou moins) - École primaire ou élémentaire (4 à 8 années d'études) - Collège ou lycée (8 à 10 ans d'études) - Lycée ou lycée (11 à 13 années d'études) - Formation technique/professionnelle (par exemple, certificat, diplôme, apprentissage) - Quelques études supérieures (collège ou université) - Diplôme de premier cycle (par exemple, baccalauréat) - Diplôme d'études supérieures (par exemple, maîtrise, doctorat) - Un niveau d'études non mentionné (veuillez préciser : ______) - Je préfère ne pas répondre | | |
| A7. SITUATION FINANCIÈRE | | | |
| Dans quelle mesure parvenez-vous facilement à payer vos dépenses de base ? (Par exemple, la nourriture, le logement, les soins de santé) | - Peut se le permettre très facilement - Peut se le permettre facilement - Je peux à peine me le permettre - Je ne peux pas me le permettre - Je préfère ne pas répondre | | |
| A8. STATUT DE TRAVAIL | | | |
| Qu’est-ce qui décrit le mieux votre situation de travail rémunéré actuelle ?  ** NOTE :*  *Inclure une question supplémentaire* | - Travailleur à temps plein - Travailleur à temps partiel * - Ne fonctionne pas * - Une situation de travail non répertoriée (veuillez préciser : ___ _) * - Je préfère ne pas répondre   *Sélectionnez les raisons de votre situation professionnelle que vous souhaitez fournir :  *Sélectionnez tout ce qui s'applique.*   - - Handicap ou raisons de santé   - Parent/responsable à domicile/aidant « à la maison »   - Étudiant   - À la retraite   - Impossible de trouver du travail   - Une raison non mentionnée (veuillez préciser : ____)   - Pas pertinent/je préfère ne pas répondre | | |

^un^ manuscrit « Explication et élaboration » de l' ISSHOO est en cours

**Appendix 15: ISSHOOs Set A – Russian***

*Note: This translation has been machine-generated using Google Translate. A validated translation is being developed and will be available on the ISSHOOs website when completed ([www.isshoos.org](http://www.isshoos.org))

*Примечание: Этот перевод был сгенерирован машиной с помощью Google Translate. Утвержденный перевод находится в разработке и будет доступен на веб-сайте ISSHOOs после завершения ([www.isshoos.org](http://www.isshoos.org)

| ISSHOOs Набор A | | | |
| --- | --- | --- | --- |
| 8 пунктов, рекомендованных для включения во все исследования боли у людей с участием взрослых участников | | | |
| Целью набора A является руководство стандартизированным сбором *минимального набора социально-демографических данных,* позволяющих описать участников исследования боли по ряду характеристик, которые, как известно, влияют на здоровье и справедливость в отношении здоровья. В дополнение к набору A рассмотрите возможность включения:   - Дополнительные социально-демографические данные, имеющие отношение к условиям вашего исследования, популяции и/или исследовательским вопросам. - Подмножество предметов из набора ISSHOOs Set B.   Для всех элементов набора А :   1. При необходимости адаптируйте язык или категории ответов в соответствии с исследуемой группой/условиями. 2. Удалите все категории ответов, которые не имеют отношения к исследуемой популяции/условиям или не подходят для нее. | | | |
| Информация для участников (рекомендуется):  *Следующие вопросы запрашивают некоторую личную информацию (это обычное дело в медицинских исследованиях). Ваши ответы будут сохранены и будут представлены как «групповые ответы» — это означает, что никто не сможет связать ваши ответы с вами.*  *Вы можете быть не уверены, почему некоторые вопросы важны, или считаете, что они не имеют к вам отношения. Предоставленная вами информация, скорее всего, будет использована для составления сводки о людях, участвовавших в этом исследовании. Это также может помочь улучшить понимание того, как личные факторы, происхождение людей и их повседневная жизнь могут влиять на здоровье.* | | | |
| А1 . ВОЗРАСТ | | | |
| Ваш возраст? | - ____ (годы) - Предпочитаю не отвечать | | |
| А2.СЕКС​ | | | |
| Примечание :   1. В некоторых условиях исследования может быть наиболее целесообразным изменить или исключить преамбулу, касающуюся пола (ниже), а также изменить категории ответов по политическим, культурным, религиозным, соображениям безопасности или по другим причинам. | | | |
| Преамбула «Секс» для участников (рекомендуется):  *Пол — это ярлык (чаще всего мужской или женский), который дается ребенку при рождении на основе частей его тела. Могут быть вариации пола, например, «интерсекс», который может быть назначен при рождении или позже в жизни.* | | | |
| Какого вы пола? | - Женский - Мужской - Интерсекс - Термин не указан (укажите: ____) - Предпочитаю не отвечать | | |
| А3. ГЕНДЕРНАЯ ИДЕНТИЧНОСТЬ | | | |
| Примечание :   1. В некоторых условиях исследования может быть наиболее целесообразным изменить или исключить преамбулу и изменить категории ответов по политическим, культурным, религиозным, соображениям безопасности или по другим причинам. 2. Если исследователи решают пропустить вопрос о гендерной идентичности, мы рекомендуем включить пункт A2 (выше) и указать переменную «Пол». 3. Рассмотрите возможность предоставления более широкого спектра категорий ответов, основанных на информации, полученной от гендерно-разнообразного сообщества (имеющей отношение к участникам вашего исследования, вопросам исследования и соответствующей контексту вашего исследования). | | | |
| Преамбула для участников «Гендерная идентичность» (рекомендуется):  *Гендерная идентичность — это личное ощущение собственного пола — будь то мужчина, женщина, оба пола, ни один из них или что-то еще. Это личное, может меняться со временем и может совпадать или не совпадать с полом человека.* | | | |
| Какой(ие) термин(ы) лучше всего описывает вашу гендерную идентичность? *Выберите все подходящие варианты.* | - Женщина - Мужчина - Небинарный - Термин не указан (укажите: ____) - Я не уверен или сомневаюсь - Предпочитаю не отвечать | | |
| А4. МЕСТО ЖИТЕЛЬСТВА | | | |
| Примечание :   1. По возможности собирайте данные, соответствующие следующей глобальной классификации степени урбанизации. Адаптируйте категории ответов к условиям вашего исследования и используйте термины, соответствующие вашей исследуемой популяции. Дальнейшие указания по созданию и представлению этого элемента приведены в отдельной рукописи ^a^ .  \|  \| Краткосрочные \| Технические термины \| \| --- \| --- \| --- \| \| Уровень 1: \| Город \| Густонаселенный район \| \|  \| Город \| Большое поселение \| \| Уровень 2: \| Город и полуплотная застройка \| Зона средней плотности \| \|  \| Плотный город \| Плотное, среднее заселение \| \|  \| Полуплотный город \| Полуплотная, средняя осадка \| \|  \| Пригородная или окологородская зона \| Полуплотная зона \| \| Уровень 3: \| Сельская местность \| Малонаселенная территория \| \|  \| Деревня \| Небольшое поселение \| \|  \| Разбросанная сельская местность \| Зона низкой плотности \| \|  \| В основном необитаемая территория \| Район с очень низкой плотностью населения \|  1. Рассмотрите возможность включения дополнительных категорий ответов для сбора более подробных данных, соответствующих вашей исследуемой популяции, обстановке и целям исследования. «Сопоставьте» их с 3-уровневой классификацией (см. пример ниже). 2. Исследователи могут в качестве альтернативы или в дополнение собирать почтовые индексы участников исследования и использовать эти данные для классификации места жительства. | | | |
| Какая категория лучше всего описывает место, где вы живете? | | - Город - Город - Сельская местность - Предпочитаю не отвечать | - Неформальное поселение или городок *(Уровень 1)* - Город *(Уровень 1)*   **Example:** (South Africa)   - Город *(Уровень 2)* - Деревня *(Уровень 3)* - Сельская местность *(Уровень 3)* |
| A5. РАСА, ЭТНИЧЕСКАЯ ПРИНАДЛЕЖНОСТЬ и/или КУЛЬТУРНАЯ ИДЕНТИЧНОСТЬ | | | |
| Примечание :   1. Этот пункт позволит сообщать о расе, этнической принадлежности и/или культурной идентичности участников исследования. Мы рекомендуем создавать категории ответов, которые позволяют идентифицировать подгруппы исследуемой популяции — особенно тех, кто может испытывать маргинализацию и/или невыгодное положение. 2. Рассмотрите возможность удаления этого элемента, если вы считаете, что он может нанести серьезный ущерб или противоречит местным, региональным или национальным законам, правилам или обычаям. 3. Важно учитывать культурные нюансы:  - Адаптируйте пункт(ы) к условиям, в которых проводится исследование, и учитывайте разнообразие происхождения всей исследуемой популяции (а не только наиболее распространенных групп). - По возможности используйте термины и категории, которые были разработаны в стране, в которой проводится исследование, и считаются приемлемыми для местного населения. - Термины «раса», «этническая принадлежность» и «культурная идентичность» не являются взаимозаменяемыми. Они могут иметь разное значение и различную приемлемость в разных условиях. Используйте термины, которые наиболее подходят в вашей учебной обстановке.   Пункт A5(a): Два вопроса, рекомендуемые для исследований с участием участников из разных стран.  Пункт A5(b): Рекомендуемый подход к исследованию в одной стране или небольшом количестве выбранных стран. | | | |
| A5(a). РАСА, ЭТНИЧЕСКАЯ ПРИНАДЛЕЖНОСТЬ и/или КУЛЬТУРНАЯ ИДЕНТИЧНОСТЬ ( *МНОГОСТРАНОВОЕ* ИССЛЕДОВАНИЕ) | | | |
| Рекомендовать 2 вопроса: (i) *И* (ii) | | | |
| Вопрос (i):  В какой стране вы живете? | | - Предоставьте поле для свободного текста ответа   *ИЛИ*  Раскрывающийся список всех стран (онлайн-опрос)   - Предпочитаю не отвечать | |
| *И* Вопрос (ii):  Опишите, как вы себя идентифицируете с точки зрения расы, этнической принадлежности или культурной идентичности. | | *Укажите до 3 терминов, которые наилучшим образом описывают, как вы идентифицируете себя:*   - _________________________________ - Предпочитаю не отвечать | |
| A5(b). РАСА, ЭТНИЧЕСКАЯ ПРИНАДЛЕЖНОСТЬ и/или КУЛЬТУРНАЯ ИДЕНТИЧНОСТЬ ( исследование *по одной стране)* | | | |
| Примечание :   1. Предпочтительный подход к сбору данных описан ниже. 2. Конкретные для каждой страны рекомендации и примеры можно найти на [сайте www.isshoos.org](http://www.isshoos.org) . Использование приведенных примеров рекомендуется для упрощения стандартизации сбора данных и отчетности. 3. Наиболее целесообразным может оказаться предоставление возможности дать ответ в виде свободного текста — см. Вопрос (ii) (выше). | | | |
| Какая категория лучше всего описывает вашу самоидентификацию? | *Разработайте категории ответов, соответствующие конкретным условиям исследования.*   - *Соберите как можно более подробную информацию о расе, этнической принадлежности и/или культурной идентичности.* - *Используйте термины, соответствующие и приемлемые для исследуемой группы населения. Рассмотрите возможность применения или адаптации категорий, включенных в национальные переписи населения (если таковые имеются).* - *Дальнейшие указания по созданию и представлению этого элемента приведены в отдельной рукописи «Объяснение и уточнение» ^и^ на веб-сайте ISSHOOs (* [*www.isshoos.org*](http://www.isshoos.org) *).* | | |
| А6.ОБРАЗОВАНИЕ​ | | | |
| Что из перечисленного ниже лучше всего описывает ваш наивысший уровень образования? | - Небольшое или отсутствующее формальное образование (3 года обучения или меньше) - Начальная или начальная школа (4–8 лет обучения) - Младшая средняя или средняя школа (8–10 лет обучения) - Старшая средняя школа или старшая школа (11–13 лет обучения) - Техническое/торговое/профессиональное обучение (например, сертификат, диплом, ученичество) - Высшее образование (колледж или университет) - Степень бакалавра (например, бакалавр) - Последипломное образование (например, магистр, доктор) - Уровень образования не указан (укажите: ______) - Предпочитаю не отвечать | | |
| А7. ФИНАНСОВОЕ ПОЛОЖЕНИЕ | | | |
| Насколько легко вы можете позволить себе (оплатить) основные расходы на проживание? (Например, питание, жилье, здравоохранение) | - Могу себе позволить очень легко - Могу себе позволить легко - Могу себе позволить только - Не могу себе позволить - Предпочитаю не отвечать | | |
| A8. СТАТУС РАБОТЫ | | | |
| Что лучше всего описывает вашу текущую ситуацию с оплачиваемой работой?  ** ПРИМЕЧАНИЕ :*  *Включить дополнительный вопрос* | - Постоянный работник - Неполный рабочий день * - Не работает * - Рабочая ситуация не указана (укажите: ___ _) * - Предпочитаю не отвечать   *Выберите любые причины вашей рабочей ситуации, которые вы хотели бы указать:  *Выберите все подходящие варианты.*   - - Причины инвалидности или здоровья   - «Домашний» родитель/управляющий домом/опекун   - Студент   - Ушедший на пенсию   - Невозможно найти работу   - Причина не указана (укажите: ____)   - Неактуально/предпочитаю не отвечать | | |

^а Рукопись^ ISSHOO «Объяснение и уточнение» находится в процессе

**Appendix 16: ISSHOOs Set A – Spanish***

*Note: This translation has been machine-generated using Google Translate. A validated translation is being developed and will be available on the ISSHOOs website when completed ([www.isshoos.org](http://www.isshoos.org))

*Nota: Esta traducción se generó automáticamente con Google Translate. Se está desarrollando una traducción validada que estará disponible en el sitio web de ISSHOOs una vez finalizada ([www.isshoos.org](http://www.isshoos.org)

| Conjunto A de ISSHOO | | | |
| --- | --- | --- | --- |
| 8 elementos recomendados para su inclusión en todas las investigaciones sobre el dolor humano que involucren a participantes adultos | | | |
| El objetivo del Conjunto A es guiar la recopilación estandarizada de un *conjunto mínimo de datos sociodemográficos,* lo que permite describir a los participantes en la investigación del dolor según una serie de características que influyen en la salud y la equidad sanitaria. Además del Conjunto A, considere incluir:   - Elementos sociodemográficos adicionales relevantes para su entorno de estudio, población y/o preguntas de investigación. - Un subconjunto de elementos del conjunto B de ISSHOO.   Para todos los artículos del conjunto A :   1. Adapte el lenguaje o las categorías de respuesta para que se ajusten a la población/entorno del estudio (según sea necesario). 2. Elimine cualquier categoría de respuesta que no sea relevante o apropiada para la población/entorno del estudio. | | | |
| Información para los participantes (recomendado):  *Las siguientes preguntas solicitan información personal (esto es habitual en la investigación sanitaria). Sus respuestas se mantendrán seguras y se registrarán como "respuestas grupales", lo que significa que nadie podrá vincularlas con usted.*  *Quizás no esté seguro de la importancia de algunas preguntas o piense que no son relevantes para usted. Es probable que la información que proporcione se utilice para elaborar un resumen de las personas que participaron en esta investigación. También puede ayudar a comprender mejor cómo los factores personales, los antecedentes y la vida cotidiana de las personas pueden influir en la salud.* | | | |
| A1 . EDAD | | | |
| ¿Cuál es tu edad? | - ____ (años) - Prefiero no responder | | |
| A2. SEX | | | |
| Nota :   1. En algunos entornos de estudio puede ser más apropiado alterar o excluir el preámbulo de sexo (a continuación) y modificar las categorías de respuesta debido a razones políticas, culturales, religiosas, de seguridad u otras. | | | |
| Preámbulo de 'Sexo' para los participantes (recomendado):  *El sexo es una etiqueta (generalmente masculino o femenino) que se le asigna a un bebé al nacer, según las partes de su cuerpo. Puede haber variaciones en el sexo, como la «intersexualidad», que puede asignarse al nacer o más adelante.* | | | |
| ¿Cual es tu sexo? | - Femenino - Masculino - Intersexual - Un término no listado (especifique: ____) - Prefiero no responder | | |
| A3. IDENTIDAD DE GÉNERO | | | |
| Nota :   1. En algunos entornos de estudio puede ser más apropiado alterar o excluir el preámbulo y modificar las categorías de respuesta debido a razones políticas, culturales, religiosas, de seguridad u otras. 2. Cuando los investigadores deciden omitir la pregunta sobre la identidad de género, recomendamos incluir el ítem A2 (arriba) e informar la variable “Sexo”. 3. Considere proporcionar una gama más amplia de categorías de respuestas informadas por la comunidad con diversidad de género (relevantes para los participantes de su estudio, las preguntas de investigación y según sea apropiado en el contexto de su estudio). | | | |
| Preámbulo de 'Identidad de género' para los participantes (recomendado):  *La identidad de género es la percepción personal del propio género, ya sea hombre, mujer, ambos, ninguno o cualquier otra. Es personal, puede cambiar con el tiempo y puede o no coincidir con el sexo de una persona.* | | | |
| ¿Qué término(s) describen mejor tu identidad de género? *Seleccione todas las que correspondan.* | - Mujer - Hombre - No binario - Un término no listado (especifique: ____) - No estoy seguro, o tengo dudas - Prefiero no responder | | |
| A4. LUGAR DE RESIDENCIA | | | |
| Nota :   1. Siempre que sea posible, recopile datos que concuerden con la siguiente clasificación global del grado de urbanización. Adapte las categorías de respuesta al entorno de su estudio y utilice términos relevantes para la población de estudio. Se proporciona más orientación para la generación y el informe de este elemento en un manuscrito aparte ^.^  \|  \| Términos cortos \| Términos técnicos \| \| --- \| --- \| --- \| \| Nivel 1: \| Ciudad \| Zona densamente poblada \| \|  \| Ciudad \| Gran asentamiento \| \| Nivel 2: \| Ciudad y zona semidensa \| Área de densidad intermedia \| \|  \| Ciudad densa \| Asentamiento denso y medio \| \|  \| Ciudad semidensa \| Asentamiento semidenso y medio \| \|  \| Área suburbana o periurbana \| Área semidensa \| \| Nivel 3: \| Zona rural \| Zona escasamente poblada \| \|  \| Aldea \| Pequeño asentamiento \| \|  \| Zona rural dispersa \| Zona de baja densidad \| \|  \| Zona mayoritariamente deshabitada \| Zona de muy baja densidad \|  1. Considere incluir categorías de respuesta adicionales para recopilar datos más granulares, relevantes para la población de estudio, el entorno y los objetivos de la investigación. Asignarlas a la clasificación de 3 niveles (ver ejemplo a continuación). 2. Los investigadores pueden, alternativamente o además, recopilar el código postal de los participantes de la investigación y utilizar estos datos para clasificar el lugar de residencia. | | | |
| ¿Qué categoría describe mejor el lugar donde vives? | | - Ciudad - Ciudad - Zona rural - Prefiero no responder | - Asentamiento informal o municipio *(Nivel 1)* - Ciudad *(Nivel 1)*   **Example:** (South Africa)   - Ciudad *(Nivel 2)* - Aldea *(Nivel 3)* - Zona rural *(Nivel 3)* |
| A5. RAZA, ETNICIDAD y/o IDENTIDAD CULTURAL | | | |
| Nota :   1. Este elemento permitirá reportar la raza, etnia y/o identidad cultural de los participantes del estudio. Recomendamos generar categorías de respuesta que permitan identificar subgrupos de la población del estudio, en particular aquellos que puedan experimentar marginación y/o desventaja. 2. Considere eliminar este artículo si cree que causará una ofensa grave o si contradice leyes, regulaciones o costumbres locales, regionales o nacionales. 3. Es importante ser sensible a los matices culturales:  - Adapte los elementos al entorno en el que se lleva a cabo el estudio y considere la diversidad de orígenes de toda la población del estudio (no solo de los grupos más comunes). - Siempre que sea posible, utilice términos y categorías que se hayan derivado en el país en el que se realiza el estudio y que la población local considere aceptables. - Los términos «raza», «etnicidad» e «identidad cultural» no son intercambiables. Pueden tener diferentes significados y diversa aceptabilidad en distintos contextos. Utilice los términos más apropiados para su contexto de estudio.   Punto A5(a): Dos preguntas recomendadas para investigaciones que involucran a participantes de varios países.  Punto A5(b): Enfoque recomendado para la investigación en un solo país o en un pequeño número de países seleccionados. | | | |
| A5(a). RAZA, ETNIA y/o IDENTIDAD CULTURAL ( Estudio *multipaís )* | | | |
| Recomendar 2 preguntas: (i) *Y* (ii) | | | |
| Pregunta (i):  ¿En qué país vives? | | - Proporcionar un cuadro de respuesta de texto libre   *O*  Una lista desplegable de todos los países (encuesta en línea)   - Prefiero no responder | |
| *Y* Pregunta (ii):  Describe cómo te identificas en términos de raza, etnia o identidad cultural. | | *Proporcione hasta 3 términos que describan mejor cómo se identifica:*   - _________________________________ - Prefiero no responder | |
| A5(b). RAZA, ETNIA y/o IDENTIDAD CULTURAL ( Estudio de *un solo* país) | | | |
| Nota :   1. A continuación se describe el enfoque preferido para la recopilación de datos. 2. Se pueden encontrar directrices y ejemplos específicos para cada país en [www.isshoos.org](http://www.isshoos.org) . Se recomienda utilizar los ejemplos proporcionados para facilitar la estandarización de la recopilación y la presentación de datos. 3. Tal vez se considere más apropiado ofrecer una opción de respuesta de texto libre: consulte la pregunta (ii) (arriba). | | | |
| ¿Qué categoría describe mejor cómo te identificas? | *Desarrollar categorías de respuesta relevantes para el entorno de estudio específico.*   - *Recopilar información sobre raza, etnia y/o identidad cultural con el mayor detalle posible.* - *Utilice términos relevantes y aceptables para la población de estudio. Considere aplicar o adaptar las categorías incluidas en las encuestas del Censo Nacional (si están disponibles).* - *Se proporciona orientación adicional para la generación y el informe de este elemento en un manuscrito separado titulado 'Explicación y elaboración' ^y^ en el sitio web de ISSHOOs (* [*www.isshoos.org*](http://www.isshoos.org) *).* | | |
| A6. EDUCACIÓN | | | |
| ¿Cuál de las siguientes opciones describe mejor su nivel más alto de educación? | - Poca o ninguna escolaridad formal (3 años de educación o menos) - Escuela primaria o elemental (4 - 8 años de educación) - Secundaria inferior o secundaria (8 a 10 años de educación) - Secundaria superior o preparatoria/bachillerato (11 a 13 años de educación) - Formación técnica/comercial/profesional (por ejemplo, certificado, diploma, aprendizaje) - Algunos estudios superiores (universidad o colegio) - Título universitario de grado (por ejemplo, licenciatura) - Título de posgrado (p. ej. maestría, doctorado) - Un nivel de educación no enumerado (especifique: ______) - Prefiero no responder | | |
| A7. SITUACIÓN FINANCIERA | | | |
| ¿Con qué facilidad puede afrontar sus gastos básicos de vida (por ejemplo, comida, vivienda, atención médica) ? | - Se lo puedo permitir muy fácilmente - Se lo puedo permitir fácilmente - Sólo puedo permitírmelo - No se puede permitir - Prefiero no responder | | |
| A8. ESTADO LABORAL | | | |
| ¿Qué es lo que mejor describe su situación laboral remunerada actual?  ** NOTA :*  *Incluir pregunta adicional* | - Trabajador a tiempo completo - Trabajador a tiempo parcial * - No funciona * - Una situación laboral no listada (especifique: ___ _) * - Prefiero no responder   *Seleccione cualquier motivo de su situación laboral que le gustaría proporcionar:  *Seleccione todas las que correspondan.*   - - Razones de discapacidad o salud   - Padre/administrador del hogar/cuidador 'en casa'   - Alumno   - Jubilado   - No puedo encontrar trabajo   - Un motivo no mencionado (especifique: ____)   - No relevante/prefiero no responder | | |

^a^ El manuscrito 'Explicación y elaboración' de ISSHOO está en proceso
